# Scaling Up Synthetic Cell Production Using Robotics and Machine Learning Toward Therapeutic Applications

**DOI:** 10.1002/adbi.202400671

**Published:** 2025-03-31

**Authors:** Noga Sharf‐Pauker, Ido Galil, Omer Kfir, Gal Chen, Rotem Menachem, Jeny Shklover, Avi Schroeder, Shanny Ackerman

**Affiliations:** ^1^ The Louis Family Laboratory for Targeted Drug Delivery and Personalized Medicine Technologies Department of Chemical Engineering Technion – Israel Institute of Technology Haifa 32000 Israel; ^2^ The Norman Seiden Multidisciplinary Program for Nanoscience and Nanotechnology Technion – Israel Institute of Technology Haifa 32000 Israel; ^3^ Faculty of Computer Science Technion – Israel Institute of Technology Haifa 32000 Israel; ^4^ The Interdisciplinary Program for Biotechnology Technion – Israel Institute of Technology Haifa 32000 Israel; ^5^ Cell Biology and Cancer Science Rappaport Faculty of Medicine Technion – Israel Institute of Technology Haifa 32000 Israel

**Keywords:** AI, artificial Intelligence, automation, machine learning, robotics, synthetic biology, synthetic cells

## Abstract

Synthetic cells (SCs), developed through bottom‐up synthetic biology, hold great potential for biomedical applications, with the promise of replacing malfunctioning natural cells and treating diseases with spatiotemporal control. Currently, most SC synthesis and characterization processes are manual, limiting scalability and efficiency. In this study, an automated method is developed for large‐scale production of protein‐producing SCs for therapeutic applications. The optimized process, compatible with a robotic liquid handling system (LiHa), reduces production time by half. Additionally, incorporation of an automated tissue dissociator‐based emulsification increases batch size 30‐fold while preserving SC characteristics. To assess SC quality and protein synthesis, artificial intelligence (AI)‐based image analysis is employed, allowing for automated, accurate and high‐throughput SC characterization. Large‐scale luciferase‐expressing SCs from a single homogeneous batch are administered to mice, allowing for real‐time monitoring of protein expression and reducing experimental variability. By troubleshooting several central steps in SC synthesis, it is demonstrated that automation and computerized quality control can significantly improve the process of SC synthesis for preclinical and clinical applications.

## Graphical abstract

Synthetic cells (SCs) hold great promise for biomedical applications, but manual production limits scalability. This study presents an automated method for large‐scale SC synthesis, integrating robotic liquid handling and machine learning‐driven high‐throughput characterization. The optimized process improves efficiency, increases batch size 30‐fold, and ensures uniformity, advancing SC applications in preclinical and clinical research.

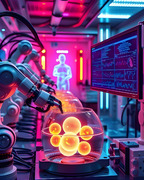

## 1 Introduction

The therapeutic potential of synthetic cells (SCs) has increasingly attracted the attention of biomedical engineers, offering novel therapeutic and diagnostic applications. SCs are microparticles designed from the bottom up to mimic the complex processes, structures, and functions of living cells while introducing functionalities beyond the capabilities of natural systems.^[^ ^1^, ^2^, ^3^, ^4^, ^5^, ^6^, ^7^ ^]^ Living cells exhibit remarkable abilities, such as directed localization, responsive behaviors, gene expression, and metabolic functions, many of which SCs aim to replicate or enhance in theranostic applications. However, to fully harness the potential of SCs for biomedical use, it is essential to develop standardized production methods, ensure high throughput, minimize toxicity, and address challenges related to long‐term storage.^[^ ^8^ ^]^

SCs serve as a bridge between bio‐inspired engineering and medicine. Constructed in the lab, these encapsulated bioreactors perform metabolic reactions and other life‐like functions using non‐living components, synthetic membranes, and synthetically engineered genomes.^[^ ^1^, ^4^ ^]^ SCs not only reproduce natural biological processes like protein synthesis,^[^ ^9^, ^10^ ^]^ ATP production,^[^ ^2^, ^11^, ^12^ ^]^ cytoskeleton re‐arrangement^[^ ^13^, ^14^ ^]^ and DNA replication,^[^ ^15^ ^]^ but also integrate novel synthetic elements such as bioluminescent intracellular signaling,^[^ ^16^ ^]^ polymeric membranes,^[^ ^17^ ^]^ and DNA‐based or lipid‐based artificial membrane receptors.^[^ ^18^, ^19^, ^20^, ^21^, ^22^ ^]^ As therapeutic platforms, SCs offer a range of advantages that go beyond those of living cells, owing to their complete engineerability and adaptable design options. The controllability and programmability of SCs, including their size, activity, internal composition, and membrane properties, hold significant promise for various clinical applications.^[^ ^9^, ^16^, ^23^, ^24^, ^25^ ^]^ Examples include insulin‐secreting synthetic beta cells,^[^ ^26^, ^27^ ^]^ toxin‐producing SCs to target and eliminate 4T1 breast cancer cells,^[^ ^28^ ^]^ and growth factor‐producing SCs that support tissue remodeling and regeneration processes.^[^ ^29^ ^]^

Therapeutic applications, however, depend on the scaling up of SC production. Unlike smaller liposome drug delivery vehicles, synthetic cells are considerably larger (single microns vs tens of nanometers in diameter) and contain a diverse array of enzymes that cannot be encapsulated using conventional methods employed for liposomal drugs.^[^ ^4^ ^]^ Current production techniques usually produce SCs in low volumes—from tens to hundreds of microliters at a time. However, therapeutic applications require standardized production processes capable of reproducibly generating homogenous SCs to meet safety measure and regulatory requirement.^[^ ^4^, ^30^, ^31^ ^]^ The limited quantities of SCs may pose challenges for future clinical applications.

Moreover, the preparation and storage of stock solutions present a significant challenge in SC production, particularly concerning time consumption and manual labor.

**Figure 1:**
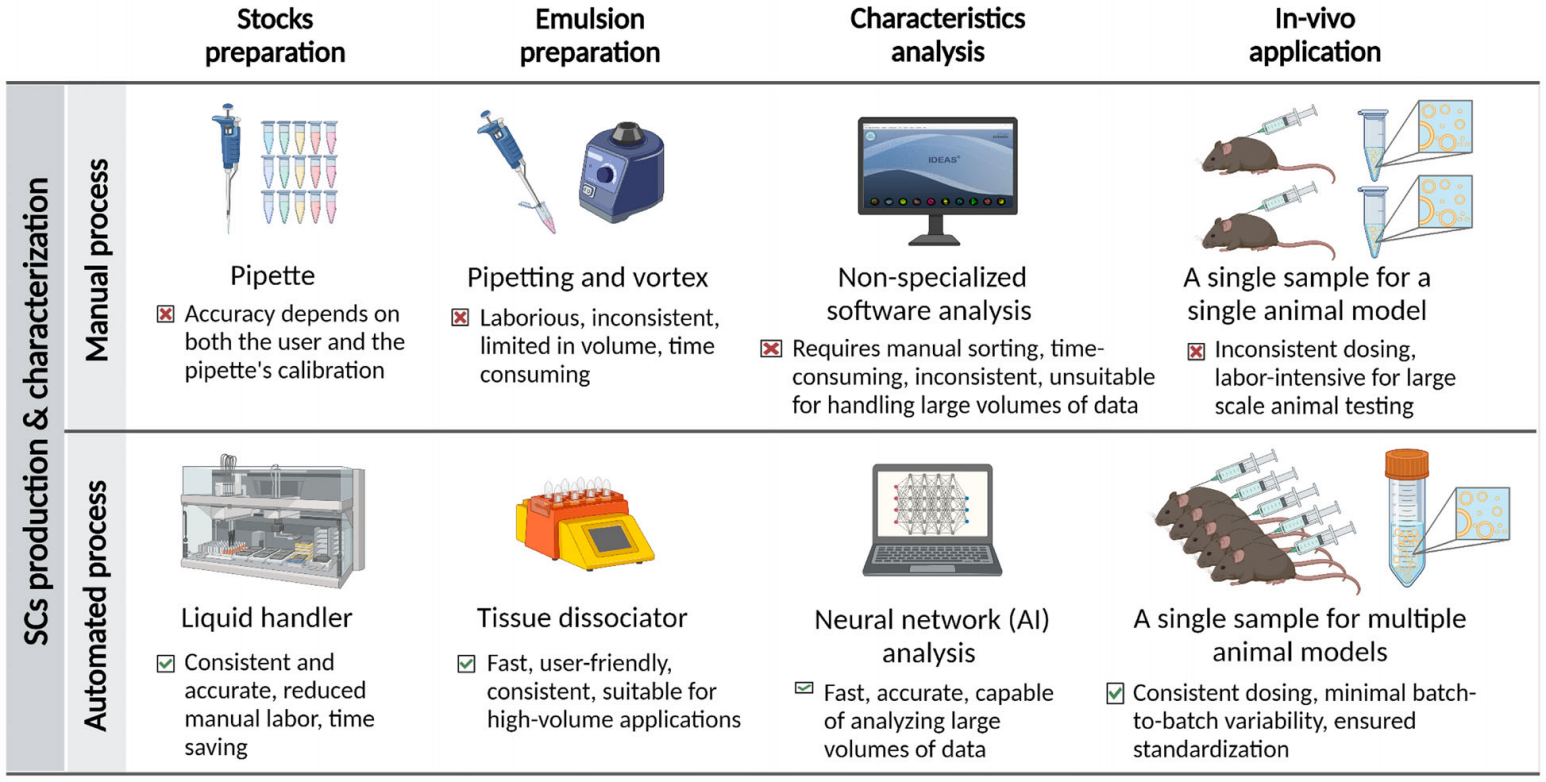
Advantages of automation in synthetic cell preparation.

**Figure 2:**
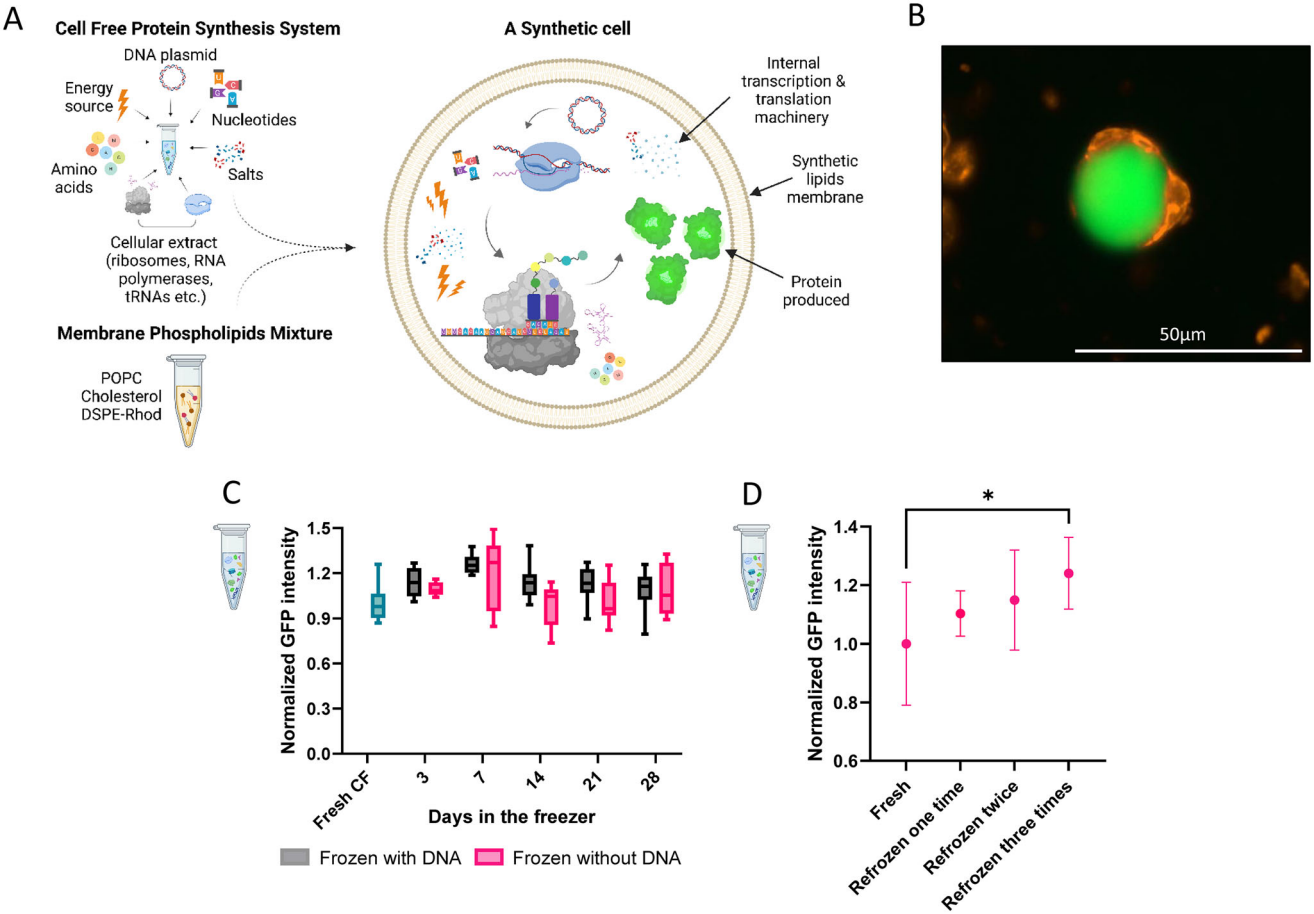
Preparation and storage of stock solutions comprising the cell‐free protein synthesis (CFPS) system for synthetic cells (SCs) production. A) A schematic illustration of protein producing SCs composition ‐ CFPS system solution encapsulated inside synthetic phospholipids membrane composed of POPC and cholesterol in 1:2 molar ratio, 1:1 volume ratio, respectively. B) A representative image of sfGFP‐producing SC after 2 h of expression at 37 °C; produced GFP (in green), Rhodamine–labeled membrane (in orange). The image was taken using a fluorescent microscope with a RFP and a GFP filters, merged. C) sfGFP production in a CFPS reaction supplemented with freshly‐made or stored in the freezer (−20 °C) pre‐inner mix solution for up to 28 days. Pre‐inner mix solution was prepared and frozen with or without the addition of DNA plasmid. Data are expressed as mean ± s.e.m. Two‐way ANOVA with adjusted P value in Tukey multiple comparisons tests, P=>0.1234 (n = 5 independent samples). D) sfGFP production in a CFPS reaction supplemented with fresh or one to three times refrozen (−20 °C) in‐house‐produced E.coli S30 lysate solution. Data are expressed as mean ± s.e.m. Two‐way ANOVA with adjusted P‐value in Dunnett multiple comparisons tests, *P = 0.0332 (n = 6 independent samples).

In this study, we introduce an automated method for producing SCs in high volumes, coupled with SC characterization using machine learning techniques (the main findings of this study are outlined in **Figure** **1** and **Table** **1**). These SCs consist of giant unilamellar vesicles (GUVs) encapsulating a bacterial‐based cell‐free protein synthesis (CFPS) system to enable protein production (**Figure** **2**A,B). We automated SC solution assembly using a liquid handling system (LiHa) and optimized the production process to minimize manual labor and time. Additionally, we refined the data analysis by incorporating convolutional neural network (CNN) to assess SC characteristics. Initial manual attempts to scale up production volumes have shown promising results but also highlighted difficulties in maintaining quality and activity levels. Subsequently, we explored automated emulsification using a tissue dissociator, which employs mechanical shear forces through rotational and oscillatory movements to break down structures while preserving viability. This method yielded consistent results across various volumes, enabling the generation of up to 24 mL of SCs in a single run. We then conducted a proof‐of‐concept experiments administering Nano‐luciferase expressing SCs to mice from a single batch, allowing us to monitor in situ protein production over time and evaluate variability between test subjects. These experiments underscore the ability to scale‐up SC production for pre‐clinical biomedical applications.

**Table 1:** The main findings of this study.

Main findings	Description and significance
Preparation and Storage of Stock Solutions	The CFPS inner solution, feeding solution, lipid solutions, and in‐house bacterial lysate can be stored at −20 °C and repeatedly thawed without compromising SC activity. This practice supports efficient batch production and reduces variability.
Automation of Stock Solution Preparation	Implementation of a liquid handling system (LiHa) to automate stock solution preparation, increasing consistency and reducing user variability, time consumption and manual labor in SC production.
Reducing Oil Droplets and Streamlining Work Time	Optimization of the SC production process by reducing lipid mixture volume and shortening pipetting and vortexing steps, significantly increasing SC concentration and activity while minimizing manual labor and oil droplet remnants.
Scaling‐Up SC Production	Development of an automated, tissue dissociator‐based emulsification method for large‐scale SC production, while maintaining consistent SC characteristics. An in vivo demonstration of Nano‐luciferase‐expression in mice from a single SC batch further highlights the low variability achieved through this automated process.
AI‐Based SC Characterization	Application of a CNN for rapid and precise high‐throughput SC characterization. The CNN‐based classification demonstrates improved accuracy over non‐specialized software classification, particularly in distinguishing SCs from oil droplets.

## 2 Results and Discussion

### 2.1 Preparation and Storage of Stock Solutions

The preparation of bacterial‐based SC systems—comprising *E. coli* S30 lysate, CFPS solution, feeding solution, and lipid mixture—has been subjected to variability in accuracy and consistency, influenced mainly by operator proficiency and the complexity of multiple procedural steps (see the protocol in Supplementary S1 and Filmed Protocol, and Materials and Methods in Supplementary S2, Supporting Information).^[^ ^16^, ^28^, ^32^ ^]^

Utilizing super‐folder green fluorescent protein (sfGFP)‐encoding DNA template, we demonstrate that both the CFPS pre‐inner solution (the CFPS inner solution excluding *E.coli* S30 lysate) and the feeding solution can be prepared in advance and stored as stocks at −20 °C for extended durations (Figure 2C). Similarly, the two lipid stock solutions used (POPC and cholesterol in mineral oil) remained stable for at least one month at −20 °C, preserving SC concentration and activity. These lipid solutions, along with the CFPS inner solution and feeding solutions, can be repeatedly thawed and refrozen without affecting CFPS and SC characteristics (Supplementary S2 Figure S1, Supporting Information). Notably, despite containing a broad range of proteins, our in‐house prepared *E.coli* S30 lysate can undergo repeated freeze‐thaw cycles at −20 °C without compromising its potency (Figure 2D).

The ability to store and refreeze large volumes of stock solutions enhances consistency and minimizes waste in SC production, which is essential for maintaining precision in therapeutic applications.

### 2.2 Automation of Stock Solution Preparation

**Figure 3:**
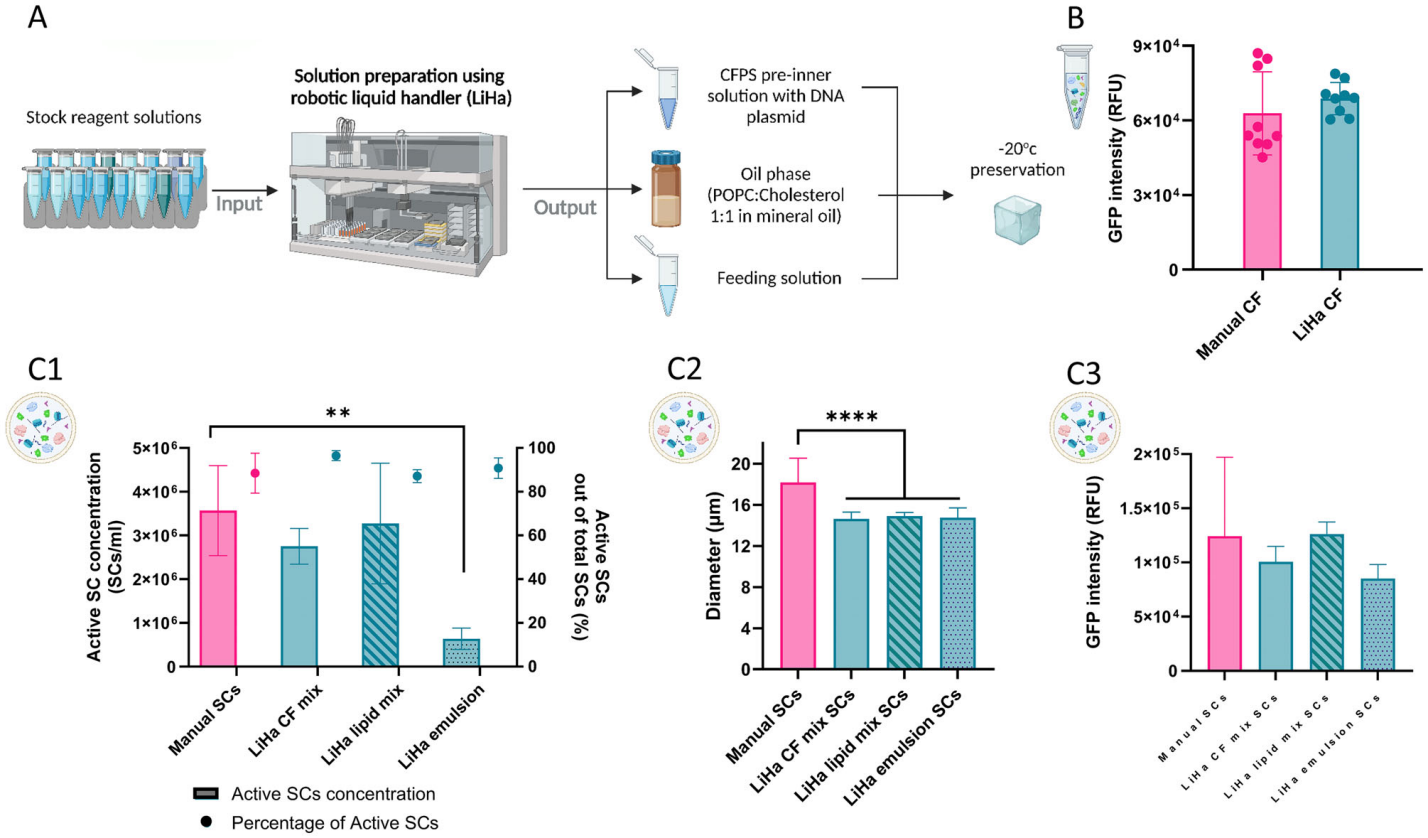
Liquid handler (LiHa) utilization for the automated preparation of SC solutions. A) Schematic illustration of LiHa usage in the preparation of SC solutions. B) Characterization of CFPS solutions prepared using LiHa compared to manually produced solutions. Unpaired two‐tailed t test P value, P = > 0.1234 (n = 9 independent samples). C) Neural network characterization of SC populations created using LiHa at various steps of the process, compared to fully manual production. This includes the preparation of pre‐inner solution, lipid mix in mineral oil, and emulsification. 1) Active SC concentration and active SC percentage. Data are expressed as mean ± s.e.m. Two‐way ANOVA with adjusted P value in Tukey multiple comparisons tests, P = > 0.1234, **P = 0.1234 (n = 6 independent samples).

**Figure 4:**
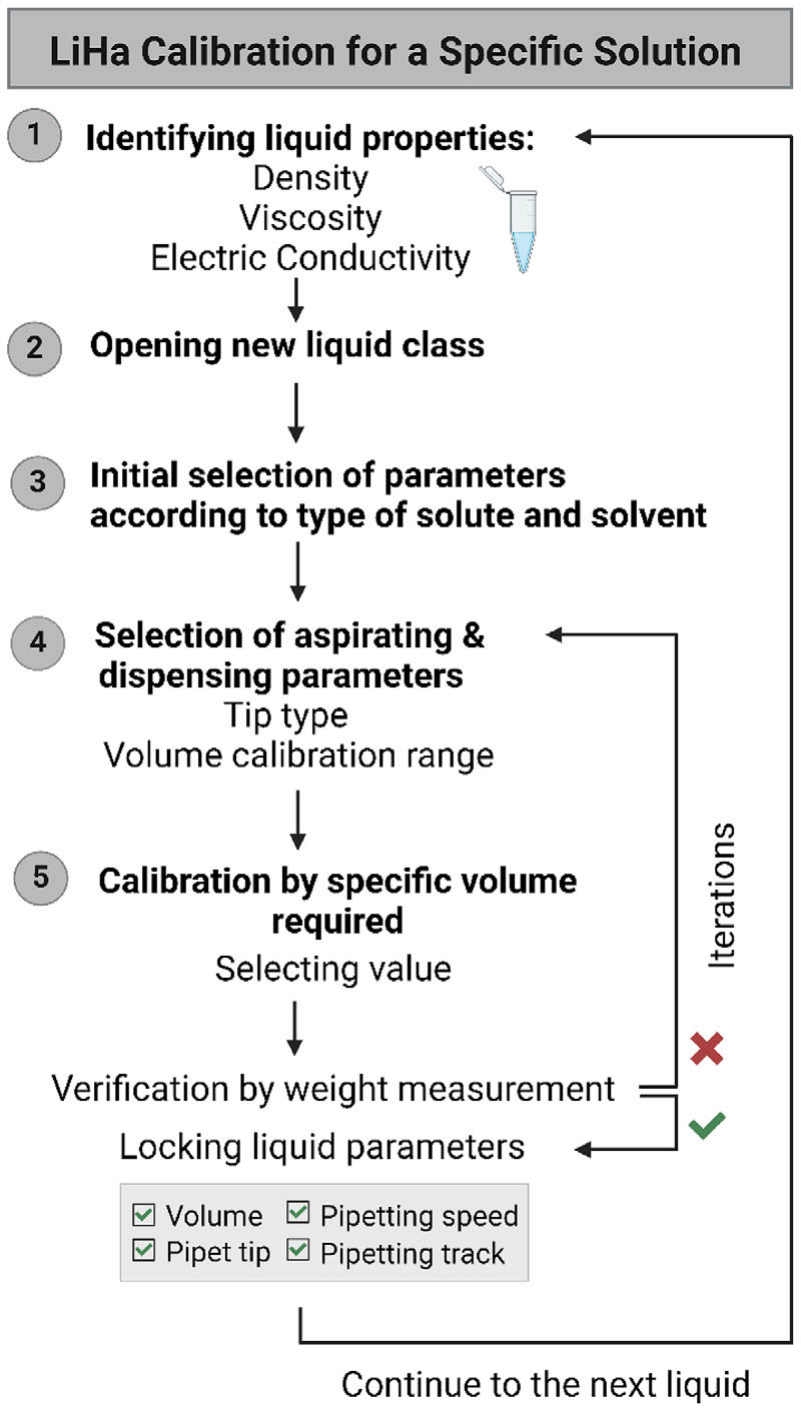
Conceptual calibration process of the robotic LiHa for specific liquid types.

We implemented an automated liquid handling system (LiHa) (Freedom EVO 75, Tecan) for the preparation of the CFPS pre‐inner solution and lipid mixture solution used in SC production (**Figures** **3**A and **4**; Supplementary S3, Supporting Information). LiHas allows for high‐precision handling of multi‐component liquids, and can be customized for different solution types.^[^ ^33^ ^]^ To further streamline SC production, emulsification was also automated with the LiHa (scripts for all steps are provided in Supplementary S3, Supporting Information).

We first compared the protein production capacity of CFPS inner solutions prepared manually and by LiHa. While both methods yielded comparable levels of sfGFP, the samples prepared using LiHa exhibited greater consistency (Figure 3B).

Next, we produced SCs using the LiHa, where each sample utilized the LiHa system for a specific step—either for preparing one of the solutions or for emulsification—enabling us to compare their characteristics with those of manually prepared SCs (Figure 3C(1–3)).

Notably, SCs produced using LiHa‐prepared CFPS pre‐inner or lipid mixture solutions achieved similar particle concentrations and percentages of active SCs compared to those prepared manually. However, LiHa‐emulsified SCs exhibited significantly lower particle concentrations (Figure 3C1) and longer emulsification times (25 min) than manually prepared samples (2 min). Despite being smaller in size (15.4±0.1 µm compared to 18.2±0.9 µm for all‐manually SCs, Figure 3C2), the LiHa‐prepared SCs maintained comparable protein production capacities, with a slight decrease in activity observed for the LiHa‐emulsified SCs (Figure 3C3).

Overall, LiHa demonstrated effectiveness in preparing solutions for SC production but was less efficient in emulsification. By reducing human error during solution assembly, this automated system enhances reproducibility and reliability, thereby increasing the applicability of the final product.

### 2.3 Reducing Oil Droplets and Streamlining SC Production Time

**Figure 5:**
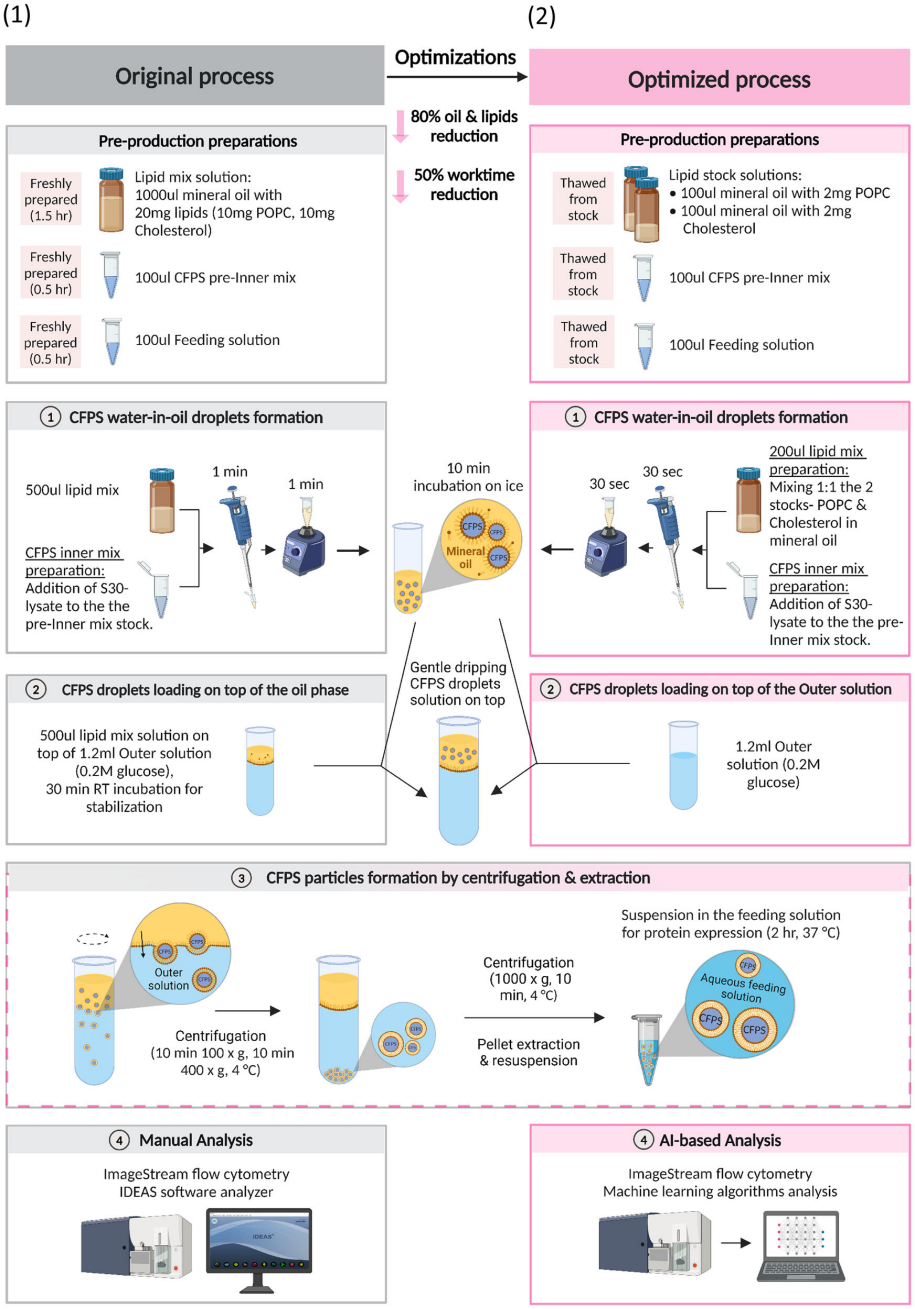
A schematic illustration comparing the original SC preparation process (1) with the optimized process (2). In short, the lipid mixture to CFPS inner solution ratio was reduced from 1:5 to 1:2, while pipetting and vortexing times were halved (step 1). Step 2 was shortened by omitting its initial phase. Additionally, SCs were analyzed using a CNN instead of non‐specialized analysis software (step 4).

**Figure 6:**
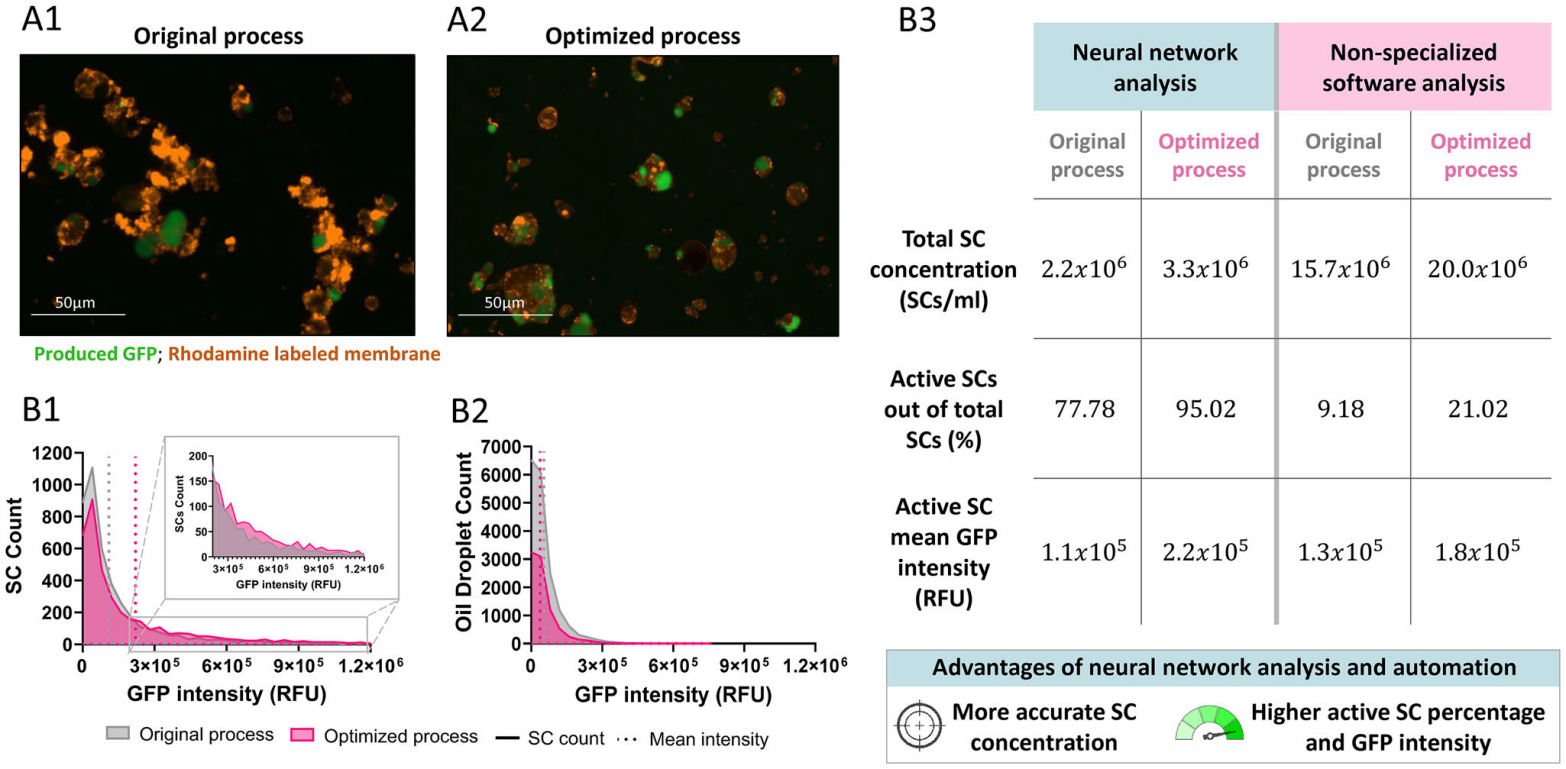
Optimization of the SC production process. A) Representative images of sfGFP‐producing SCs generated through the original (1) or the optimized process (2), after 2 h of protein expression at 37 °C; produced GFP (in green), Rhodamine –labeled membrane (in orange). The images were captured using a fluorescent microscope with a RFP and a GFP filters, merged. B) Neural network characterization of particle populations generated by the original process compared to those produced by the optimized process. 1) Characterization of the sfGFP‐producing SC population. 2) Characterization of the oil droplet population. 3) Comparison of SC characteristics to non‐specializedsoftware analysis, including total SC concentration, percentage of active SCs, and mean sfGFP intensity.

We examined the necessity and impact of each step in the SC production process, focusing on the effects of lipid mixture‐to‐CFPS inner solution volume ratio, as well as the duration of pipetting and vortexing, on SC characteristics. Protein‐producing SCs are synthesized by encapsulating an *E. coli*‐based CFPS system within lipid vesicles^[^ ^28^, ^34^ ^]^ (the original preparation process is presented in **Figure** **5**‐1). We found that the quantity of lipid mixture solution can be reduced from 10 to 2‐times the volume of the CFPS inner solution, while maintaining SC characteristics and substantially reducing oil droplet formation in the final sample (Figures 5‐2 and **6**A,B; the protocol in Supplementary S1, Supporting Information). Additionally, we halved the duration of both the pipetting and vortex steps (from 60 to 30 seconds), reducing the manual labor involved. The optimized process led to an increase of over 50% in SC concentration (Figure 6B3). Moreover, the percentage of SCs producing sfGFP increased by over 20%, and the mean GFP intensity within the SCs doubled (from (1.1 ± 0.2)*x*10^5^RFU to (2.2 ± 0.2)*x*10^5^RFU, corresponding to $\left(1.8\pm 0.7\right)\frac{\mu g\;GFP}{ml}\;and\;\left(3.6\pm 1.4\right)\frac{\mu g\;GFP}{ml},$ respectively, Figure 6B1,B3; Supplementary S2 Figure S2, Supporting Information). These adjustments enhanced SC concentration and activity while also reducing processing time and manual labor. Furthermore, optimizing the process minimized oil residues in the samples (Figure 6B2).

Although our optimization efforts in the SC production process resulted in notable improvements in SC characteristics, some attempts were less successful. For a detailed overview of both successful and unsuccessful modifications, refer to Supplementary S4, Table S1, and Figures S1–S6.

### 2.4 Scaling‐Up SC Production

Despite significant advancements in SC production methods in recent years, current emulsion‐based methods are limited to producing SCs in small volumes, typically in the range of 100µL that are not sufficient for preclinical and clinical tests.^[^ ^16^, ^28^, ^29^, ^35^, ^36^, ^37^ ^]^ Here, we introduced an automated tissue dissociator‐based emulsification method for large‐scale SC production. The characterization and comparison of the SC samples were conducted using a neural network analysis, which will be elaborated in the following section (Figures 6B3 and 8).

**Figure 7:**
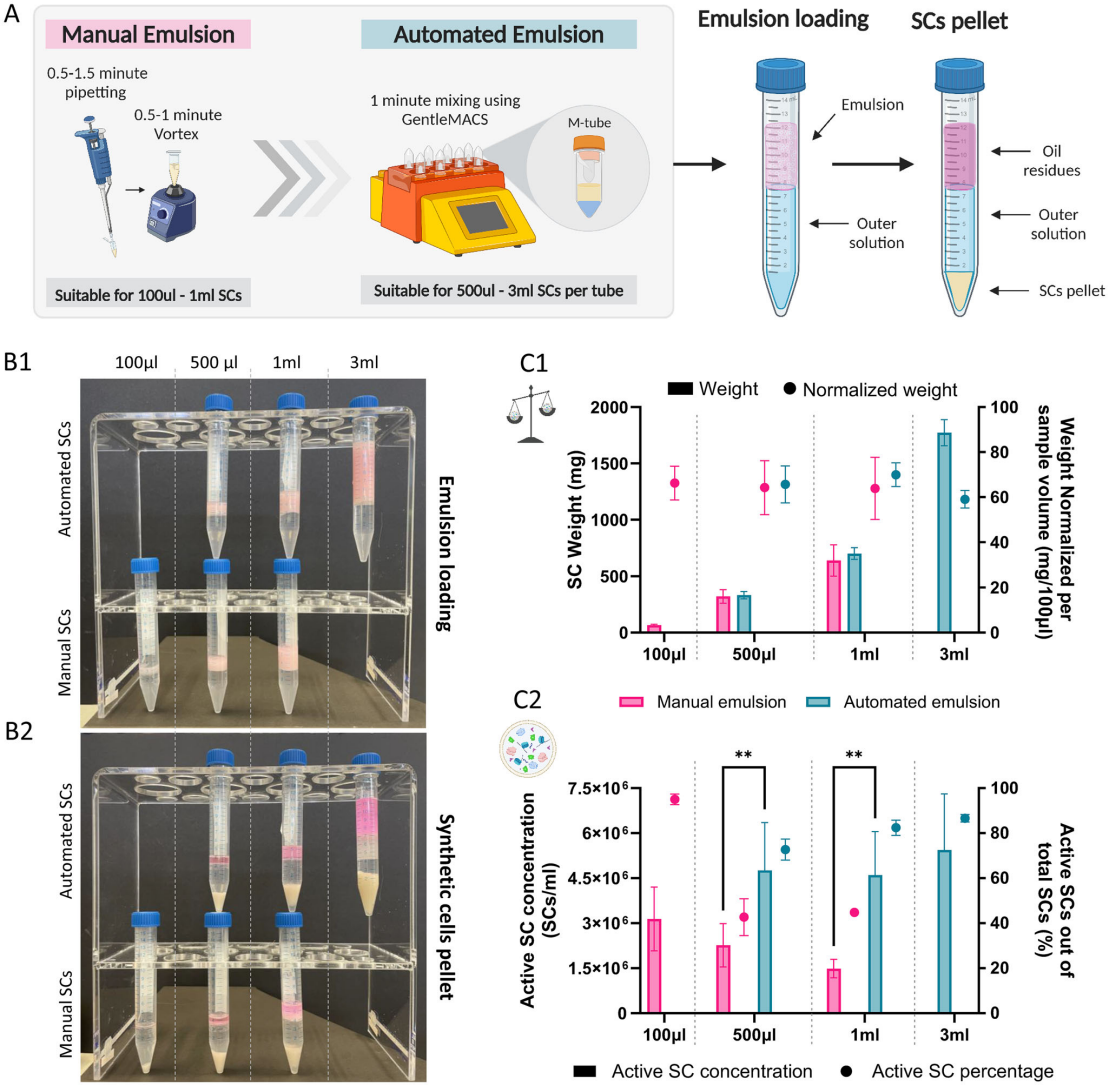
Scale‐up of SC production using manual versus automated emulsification. A) Schematic illustration of scaling up SC production through manual and automated emulsion generation processes. B) Representative images of SC emulsion loading (1) and pellet formation (2) at different emulsion volumes in both manual and automated processes. Rhodamine B dye was added to the oil phase to enhance visibility. The emulsion volume was gradually increased: manual emulsions were created using inner solution volumes of 100–1000 µL, while automated emulsions were produced with volumes ranging from 500 to 3000 µL of CFPS inner solution. C) Neural network characterization of SC population produced from each emulsion volume in the manual or automated process: 1) Total SC pellet weight and pellet weight normalized to 100 µL of CFPS solution. Data are expressed as mean ± s.e.m. Two‐way ANOVA with adjusted P value in Tukey multiple comparisons tests, P=>0.1234 (n = 4‐8 independent samples). 2) Active SC concentration and percentage. Data are expressed as mean ± s.e.m. Two‐way ANOVA with adjusted P value in Sidak's multiple comparisons t‐tests, P = >0.1234, **P = <0.0021, ****P = <0.0001 (n = 3 independent samples).

First, we manually produced SCs in CFPS volumes of 500 µL and 1 mL, compared to the initial 100µL, and found no significant difference in normalized pellet weight across all volumes tested (**Figure** **7**A,B1,B2 bottom,C1 pink). However, increasing the CFPS volume led to a decrease in active SC concentration and percentage (Figure 7C2 pink), prompting us to explore alternative automated emulsification methods (Supplementary S4 Table S1 and Figure S4, Supporting Information).

We adapted a tissue dissociator (GentleMACS, Miltenyi Biotec Inc.), typically used for generating single‐cell suspensions, to automate the SC emulsification process. This instrument provides precise control over agitation speed and duration. Our results showed that agitating the CFPS inner solution with the lipid mixture at 200 RPM for 1 min at room temperature produced an effective emulsion (see detailed protocol, Supplementary S1, Supporting Information and Filmed Protocol). To accommodate the instrument tubes' minimum volume requirement of 1.5 mL, we set the minimum CFPS inner solution volume at 500 µL, combined with 1 mL of lipid mixture. For the maximum tube capacity of 10 mL, the highest CFPS inner volume tested was 3 mL, paired with 6 mL of lipid mixture. Notably, the instrument can agitate up to eight tubes in a single run.

We then produced SCs using the tissue dissociator with CFPS inner volumes of 500 µL, 1mL, and 3mL (Figure 7B1,B2 top). As sample volumes increased, pellet weight rose proportionally, with no significant differences in mean normalized pellet weights across the samples (Figure 7C1 blue). We further examined the properties of the SCs produced through the automated emulsification. While the percentage of active SCs was slightly lower for the automated method, the automated SCs demonstrated a significantly higher concentration of active SCs compared to the manual samples (Figure 7C2 blue).

Overall, we achieved a 30‐fold increase in SC sample volume, enabling the generation of up to 24 mL of SCs in a single run while maintaining SC characteristics. Taken together, these results support the suitability of adapting tissue dissociator for scaling up the emulsion step of the process, ensuring uniform emulsification and reproducibility, and reducing batch‐to‐batch variability.

### 2.5 Neural Network‐Based SC Characterization

**Figure 8:**
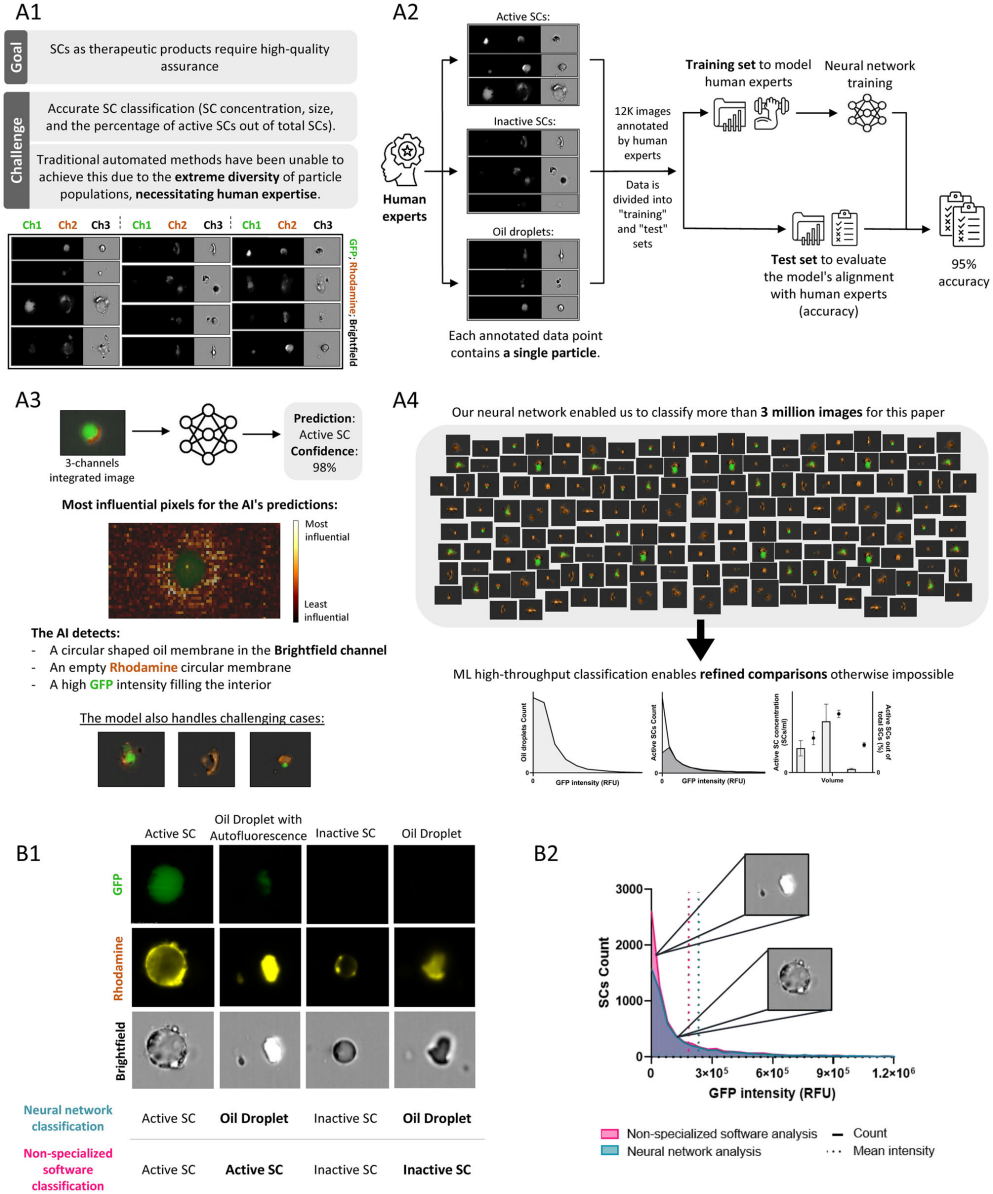
CNN‐based SC characterization. A) Machine learning (ML) for quality assurance and large‐scale SC classification: 1) The challenge of quality assurance in therapeutic SC production. 2) ML training workflow: ML models human expertise by training on annotated data. 3) Neural network predictions. 4) ML for high‐throughput classification: the trained ML model is applied for quality assurance and large‐scale SC experiments. B) Classification differences between particle identification using non‐specialized analysis software and neural network analysis of the same sample. 1) Errors in particle identification with non‐specialized analysis software compared to our neural network, based on imaging flow cytometry representative images. 2) Analysis of differences in the protein‐producing SC population generated by the optimized production process.

Scaled‐up production for therapeutic use requires constant monitoring and quality assurance, which is crucial for enabling regulation and widespread adoption of SC‐based treatments. To enhance quality assurance and enable large‐scale SC classification, we trained a convolutional neural network (CNN) (**Figure** **8**A1).

To assess large‐scale SC production, we trained a CNN, a deep neural network designed for spatial data processing, to classify active SCs, inactive SCs, and oil droplets. For training, we annotated over 10,000 imaging‐flow‐cytometry images into one of the three categories (representative images and a concise explanation of the visual features used for classification are provided in Supplementary S2 Tables S1–S5, Supporting Information). The annotated dataset was divided into training and test sets (Figure 8A2). We then manually validated the CNN results to ensure accuracy. The code for the neural network and the training data, along with running samples, are available at https://github.com/IdoGalil/syn‐cells‐classification (For further details, refer to the Experimental Section‐Neural network analysis).

CNN SC classification was compared to non‐specialized software analysis^[^ ^16^, ^28^, ^29^ ^]^ (Figures 6B3 and 8A3,B; Supplementary S2 Figures S3 and S5, Supporting Information). The traditional software classified a larger number of particles as active SCs. However, manual inspection revealed that many of these were actually oil droplets, mistakenly classified as active SCs due to their similar characteristics, such as diameter, diameter‐to‐area ratio, and fluorescent intensities (Figure 6B3; Supplementary S2 Figure S4, Supporting Information). These droplets typically have low GFP intensity, which lowers the overall mean (Figure 8B2).

For the optimized SC production process, both the mean and median GFP intensity of the active SCs are higher when classified by the neural network (Figure 6B3). The non‐specialized software reported a mean GFP intensity of (1.8 ± 0.2)*x*10^5^RFU and a median of (4.4 ± 0.8)*x*10^4^RFU, whereas the CNN's mean was (2.2 ± 0.2)*x*10^5^RFU and the median was (7.4 ± 1.0)*x*10^4^RFU. This improvement reflects the CNN's ability to analyze individual particle characteristics across a larger dataset. Supplementary S2 Figure S5 (Supporting Information) illustrates discrepancies between classification methods, highlighting the impact of these variations on particle classification.

Although in the original process the mean GFP intensity of “active SCs” characterized by the non‐specialized software is slightly higher ((1.3 ± 0.1)*x*10^5^RFU compared to (1.1 ± 0.2)*x*10^5^RFU), the median GFP intensity was lower ((1.9 ± 0.9)*x*10^4^RFU in opposed to (2.3 ± 0.8)*x*10^4^RFU). This indicates that most of the “active SCs” classified by the non‐specialized software had low GFP intensity. The higher mean GFP intensity can be attributed to a small fraction of high‐intensity oil droplets misclassified as active SCs. For example, the CNN correctly identified the highest GFP intensity of oil droplets in these samples as 7.5*x*10^5^RFU, well above the average GFP intensity of actual SCs.

For both the original and optimized processes, the neural network provided a more reliable SC count by excluding oil droplets and analyzing larger datasets. This resulted in a much higher percentage of active SCs out of the total SCs and lower total SC concentrations compared to the traditional software (Figure 6B3).

While CNN analysis does not directly alter SC production, it provides valuable insights into process optimization. By comparing SC characteristics such as active SC percentage out of the total SC population, total SC concentration, and activity across production methods, the CNN indirectly influences production processes, helping refine and streamline protocols for better outcomes. Additionally, the ability to handle large datasets enables extensive comparisons and batch analyses, ensuring higher reproducibility and robustness for future therapeutic applications (Figure 8A3,A4). These findings underscore the important role of advanced classification methods for improving the accuracy and reliability of SC characterization, enabling rapid analysis of large datasets and enhancing the potential for therapeutic applications.

### 2.6 Utilization of Scaled‐Up SC Production for Preclinical Applications

**Figure 9:**
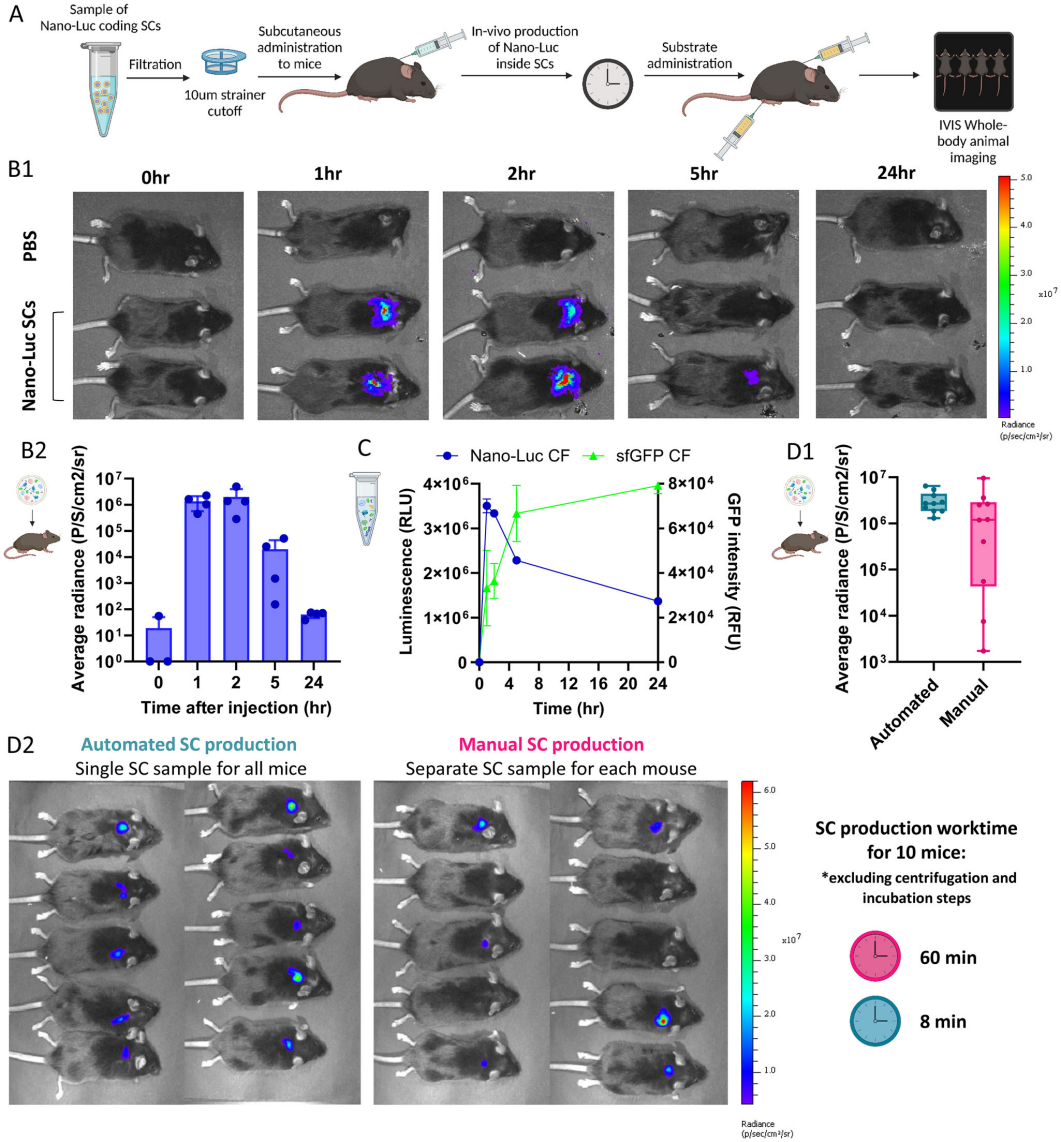
Utilization of scale‐up SC production for in‐vivo applications. A) Schematic illustration of Nano‐luciferase (Nano‐Luc)‐producing SC preparation and in‐vivo protein expression over time. B) Nano‐Luc in‐vivo production inside SCs. 1) SCs were subcutaneously injected into C57BL/6 mice (top – PBS, middle and bottom –SCs). The luminescent signal from the particles was monitored using whole‐animal imaging at 0, 1, 2, 5 and 24 h after SCs administration. Images were captured 5 min after subcutaneous administration of Nano‐Glo substrate (diluted 1:20 in PBS) via both Intraperitoneal (IP) and subcutaneous injections. 2) Average radiance of the luminescent signal (P/S/cm^2^/sr) in mice injected with Nano‐Luc‐producing SCs (n = 4 independent samples). C) The luminescent signal of Nano‐Luc‐producing CF compared to sfGFP‐producing CF over time in‐vitro (n = 3 independent samples). D) Comparison of Nano‐Luc in vivo production in SCs: Automated versus. Manual Production Methods. SCs were prepared using either the automated or manual production method and subcutaneously injected into C57BL/6 mice. Luminescent signals were monitored at peak intensity via whole animal imaging, 5 min after subcutaneous administration of Nano‐Glo substrate (1:20 dilution in PBS). 1) Average radiance of the luminescent signal (P/S/cm^2^/sr) in mice injected with Nano‐Luc‐producing SCs. 2) Whole‐animal imaging of mice injected with SCs from a single automated batch or separate manually prepared samples (n = 10 independent samples).

One of the major challenges in meeting clinical demands has been scaling up SC production.^[^ ^1^, ^4^, ^38^ ^]^ To demonstrate the scale‐up process, we conducted a proof‐of‐concept experiment involving the simultaneous administration of SCs from a single sample in a preclinical study. First, we prepared a single 3 mL formulation of Nano‐luciferase (Nano‐Luc) coding SCs using our automated scaled‐up process to evaluate in vivo protein production over time. The SCs were stored on ice before administering 150 µL subcutaneously into the backs of 20 mice, ensuring efficient in situ protein production (**Figure** **9**A,B). The luminescent signal from the SCs was monitored at 0, 1, 2, 5, and 24 h post‐administration. At each time point, four mice injected with SCs, along with a control mouse, were administered fresh substrate and sacrificed to assess the bio‐distribution of SCs across the organs (Supplementary S2 Figure S6, Supporting Information).

An increase in the luminescent signal was detected within the first hour post‐injection, indicating in vivo production of enzymatically active protein. The signal remained stable for an additional hour before gradually declining (Figure 9B1,B2).

To determine whether the decrease in luminescent values was caused by the composition or stability of the SC particles, or by their assimilation into the mice, we conducted an in vitro assessment of the luminescent signal of the Nano‐Luc‐producing CFPS sample over the same time intervals. This signal was then compared to that of the sfGFP‐producing CFPS samples. An anticipated rise in both luminescent and GFP signals was observed within the first 2 h, indicating the gradual production of Nano‐Luc and sfGFP proteins (Figure 9C). While the GFP fluorescent signal continued to rise consistently over 5 and 24 hours, the luminescent signal exhibited a similar trend to the in vivo results, decreasing over the same periods of time. The same trend was also observed in the Nano‐Luc‐producing SCs sample in vitro (Supplementary S2 Figure S7, Supporting Information). Additionally, we verified the luminescence of mice injected with Nano‐Luc‐producing SCs without Nano‐Luc substrate and SCs without Nano‐Luc DNA template with Nano‐Luc substrate to rule out any contribution to the signal from the SCs themselves or the substrate (Supplementary S2 Figure S8, Supporting Information). These results suggest that the decrease in signal is due to protein degradation rather than particle degradation.

Next, we aimed to compare the variability between test subjects using SCs produced by manual and automated processes. Minimizing heterogeneity and variability in patient responses is critical for clinical application. To evaluate our scaled‐up production process Nano‐Luc‐coding SCs were administered to two groups of ten mice: one group was subcutaneously injected with 100 µL from a single 1mL automated scaled‐up batch, while the other received 100 µL from ten manually prepared samples. The luminescent signal from the SCs was measured at peak time post‐substrate administration (Figure 9D).

Despite both groups achieving similar mean Nano‐Luc production levels, the automated group exhibited significantly lower variability, with averages of (3.1 ± 1.6)*x*10^6^ *P*/*S*/*cm* ^2^/*sr* compared to (2.1 ± 2.8)*x*10^6^ *P*/*S*/*cm* ^2^/*sr*, and relative standard deviations (calculated as standard deviation divided by the mean) of 0.5 versus 1.3, respectively.

To further assess the advantages of our automated production process, we quantified the worktime required to prepare SC samples for this in vivo study (Figure 9D2). The automated method demonstrated an ≈7.5‐fold reduction in worktime compared to the manual approach. Notably, the automated process can generate up to 3mL of SCs with minimal additional time and effort.

Together, these findings highlight the advantages of automation in producing large‐volume SC samples, including reduced worktime, minimized batch‐to‐batch variability, and enhanced scalability—critical factors for advancing SCs toward clinical applications.

## 3 Conclusion

Large‐scale production capability is crucial for ensuring consistent dosing across multiple patients, standardization and minimizing batch‐to‐batch variability. By integrating automation—specifically utilizing the LiHa for stock preparation and a tissue dissociator for emulsification—we effectively addressed several shortcomings of traditional SC production methods. The LiHa enhances consistency and accuracy in reagent dispensing, mitigating variability associated with manual pipetting. Similarly, the tissue dissociator provides a fast, user‐friendly, and reliable alternative to labor‐intensive emulsification techniques, such as pipetting and vortexing. However, integrating robotics presented both challenges and opportunities during the scale‐up. For instance, initial attempt to automate emulsification with LiHa resulted in lower SC concentrations compared to manual methods. Upon adapting the approach and employing a tissue dissociator, we achieved significant improvements, highlighting the importance of leveraging each machine's unique capabilities. Furthermore, the application of a CNN for SC evaluation demonstrated superior accuracy in classification compared to traditional methods, particularly in differentiating SCs from oil droplets. Notably, the in vivo demonstration of Nano‐luciferase expression from a single SC batch further emphasizes the low variability achieved through this automated process. Ultimately, the automated SC production method enables rapid, high‐volume output with uniform content and characteristics, advancing the potential for preclinical and clinical applications.

## 4 Experimental Section

Detailed Materials and Methods are provided in Supplementary S2 (Supporting Information).

### CF and SCs Composition

*Cell‐Free Reaction*: Cell‐free protein synthesis (CFPS) system reactions were performed using in‐house S30‐T7 bacterial lysate (mentioned in Supplementary S2, Supporting Information Materials and Methods Section 1) according to the inner solution composition detailed in Supplementary S2, Supporting Information Materials and Methods Section 2.

*Preparation of Lipid Mixture Solution for SCs Construction*: 1‐palmitoyl‐2‐oleoyl‐sn‐glycero‐3‐phosphocholine (POPC) (Lipoid, Ludwigshafen, Germany) was lyophilized (FreeZone 2.5; Labconco, USA) overnight. POPC and cholesterol (Sigma–Aldrich, Rehovot, Israel) were dissolved separately in chloroform (Bio‐Lab, Jerusalem, Israel) at a concentration of 80 mg mL^−1^ each, and then vortexed thoroughly. Subsequently, mineral oil (Sigma–Aldrich, Rehovot, Israel) was added to each solution to achieve a final lipid concentration of 20 mg mL^−1^ each. Each solution was vortexed, divided into 1 mL aliquots in Eppendorf vials, and then heated at 80 °C and 450 RPM for 1 h to evaporate the chloroform. The obtained POPC‐oil and cholesterol‐oil solutions’ aliquots were stored at ‐20 °C. For the preparation of the lipid‐oil phase for the SCs process, both lipid‐oil solutions were heated for 5 min at 37 °C and vortexed before use. Then, the solutions were mixed at a 1:1 v/v ratio in the required amount (1:2 v/v ratio of inner solution to lipids in oil mixture). Rhodamine‐labeled phospholipid (14:0 Liss Rhod PE, 1 mg mL^−1^ in ethanol) (Avanti Lipids Polar, Alabaster, AL) was incorporated by adding 0.8 µL to every 100 µL of lipid solution in mineral oil.

### SCs Preparation

*The Original SCs Preparation Process*: SCs preparation using the emulsion transfer method was performed as previously described.^[^ ^32^, ^34^ ^]^ The inner and feeding solutions for the SCs were prepared according to the composition listed in Supplementary S2 (Supporting Information) Materials and Methods Section 2 for in‐house lysate‐based SCs.

*The Optimized SCs Preparation Process*: Optimized SCs preparation using the emulsion transfer method was performed by manual emulsification for CFPS inner solution volumes of 100 µL, 500 µL, and 1 mL, and by automated emulsification using the M‐tubes of the GentleMACS tissue dissociator instrument (Miltenyi Biotec Inc., Germany) for inner solution volumes of 500 µL, 1 mL, and 3 mL, as detailed in the protocol in Supplementary S1 (Supporting Information)and Filmed Protocol.

*Automated Liquid Handler System (LiHa)*: The Tecan Freedom EVO 75 model was used for assembling solutions in SCs fabrication, including CFPS pre‐inner and lipid mixture solutions. Detailed description of LiHa calibration for each liquid type can be found in Supplementary S3 (Supporting Information).

### SCs Characterization

*Imaging Flow Cytometry Analysis and Manual Quantification (Size, Concentration, Activity, and Percentage of active SCs)*: The ImageStreamX Mk II (Luminex Corporation, USA), controlled by the AMNIS Inspire software (version 200.1.681.0) and analyzed using the IDEAS software (version 6.2), was used for this study. SCs, membrane‐labeled with rhodamine, with or without plasmid DNA encoding for sfGFP (containing an internal solution as described in Supplementary S2 (Supporting Information) Materials and Methods Sections 2 and 3), were incubated for 2 h at 37 °C and filtered with a 70 µm cell strainer (BD Biosciences, San Jose, CA) to eliminate aggregates before analysis with the ImageStreamX. The 488, 561, and 785 nm lasers were used for fluorescence excitation along with side scatter (SSC) illumination (emission band 745–780 nm). SCs events were classified using IDEAS software and manually verified using images from the brightfield and Rhodamine channels (emission band 560–595 nm). The SCs activity level, namely their protein expression capabilities, was assessed by measuring GFP fluorescence intensity using the GFP channel (emission band 505–560 nm). SCs without a DNA template served as a negative control and were used to establish the gate for GFP‐positive events. For each analyzed sample, 10 000 events were collected (n = 3). The SCs' diameter distribution, GFP intensity distribution, concentration, and percentage of active (i.e., GFP‐producing) SCs within the total SCs population were also calculated using the IDEAS software. The SCs' concentration was calculated by multiplying the overall particle concentration by the percentage of SCs in the sample.

*Neural Network Analysis*: A convolutional neural network was used as a model for a 3‐class classification: active synthetic cells, inactive synthetic cells, and oil droplets. As the architecture, EfficientNetV2‐b0 was selected due to its good performance on a validation dataset, its good speed, and its capable selective predictive performance relative to its size^[^ ^39^ ^]^ (as can be seen in the supplementary of Galil et al., 2023^[^ ^40^ ^]^). The model weights were initialized with publicly available weights pretrained on ImageNet‐1k, a standard dataset for benchmarking computer vision.^[^ ^41^ ^]^ Pretraining on ImageNet was shown to produce models that are transferable into other computer vision tasks (i.e., removing the pretrained classifier head and training the model with a new classification head on a new task).^[^ ^42^ ^]^

Images were collected from the ImageStreamX Mk II (Luminex Corporation, USA) and annotate the images into one of the three classes‐ active synthetic cells, inactive synthetic cells and oil droplets. Overall, 3.7K images of active synthetic cells, 3.6K images of inactive synthetic cells, and 4.8K images of oil droplets were annotated. Each image has three channels: GFP, Rhodamine, and Bright field. This dataset was split into a train set and a validation set in a balanced way (stratified sampling according to the label).

Since ImageNet models are trained with three color channels (RGB), each of the network's input channels were arbitrarily assigned to one of the three channels in the images (GFP, Rhodamine, Bright field). Next, the model was trained on the dataset, achieving an overall accuracy of 91% (with the accuracy per each class exceeding 90%). To further improve performance and enhance uncertainty estimates, an ensemble of 15 models were trained, boosting accuracy to 93%.^[^ ^43^ ^]^ To achieve an even higher performance, a selective accuracy constraint (SAC) of 95% was set, providing with a 93.1% coverage.^[^ ^40^ ^]^ In other words, the model only predicts for images it is confident of, so the expected accuracy will be at least 95%.

Since the dataset is imbalanced, for each epoch equal sized sets of each class were sampled. The images were resized and normalized the same way the ImageNet images were in the model's pretraining. The model for five epochs was trained with Adam as an optimizer and a Cosine Annealing scheduler and an initial learning rate of 1e‐3.^[^ ^44^ ^]^

### In‐Vivo Studies

All animal studies were approved by and complied with the institutional ethical committee at the Technion – Israel Institute of Technology (approval number IL1781123). Nano‐Luc production inside SCs in vivo was monitored using the IVIS 200 imaging system (PerkinElmer, Inc., Massachusetts, USA).

*Nano‐Luciferase (Nano‐Luc) Production In Vivo Over‐Time*: SCs with Nano‐Luc encoding DNA or without a DNA template were prepared, filtered using a 10 µm sterile syringe strainer (pluriSelect, Leipzig, DE) to reduce aggregations, and kept on ice to prevent in vitro protein production. Prior to administration, the mice were anesthetized with a mixture of Ketamine (100 mg kg^−1^) and Xylazine (10 mg kg^−1^). The prepared mixtures of protein‐producing SCs were then injected subcutaneously into the backs of eight‐week‐old female C57BL/6 mice, with each mouse receiving 150  µL of the SCs sample. Additionally, five control mice were administered PBS at the same volume. The luminescent signal from the particles was monitored using whole‐animal imaging with IVIS at 0, 1, 2, 5, and 24 h post‐administration (for each time point n = 4 for SCs, n = 1 for PBS), following mice euthanization (using Ketamine (300 mg kg^−1^) and Xylazine (30 mg kg^−1^) mixture) and Nano‐Glo substrate (Promega, United States) administration, diluted 1:20 in PBS, via both intraperitoneal injection (IP) and subcutaneous injections (4.65 µL g^−1^ each, total 150 µL mouse^−1^, 75 µL mouse^−1^ via IP injection & 75 µL mouse^−1^ via subcutaneous injection). The biodistribution of the SCs in the mice's organs was evaluated for luminescent signal using IVIS (for each time point n = 4 for SCs, n = 1 for PBS) (Supplementary S2 Figure S6, Supporting Information).

*Comparing Variability in SC Samples Produced via Automated or Manual Production Methods In Vivo*: SCs with Nano‐Luc encoding DNA were prepared, using either the automated scale‐up production method (1 sample of 1 mL) or the manual production method (10 samples of 100 µL each). The samples were kept on ice to prevent in vitro protein production. Prior to administration, the mice were anesthetized with a mixture of Ketamine (100 mg kg^−1^) and Xylazine (10 mg kg^−1^). The prepared samples of protein‐producing SCs were then injected subcutaneously into the backs of eight‐week‐old female C57BL/6 mice, with each mouse receiving 100 µL of the SC sample (n = 10 per group). Additionally, two control mice were administered PBS at the same volume. The luminescent signal from the SCs was monitored at the peak signal time (1.5 h post‐administration) using whole‐animal imaging with an IVIS system. Prior to imaging, mice were euthanized using Ketamine (300 mg kg^−1^) and Xylazine (30 mg kg^−1^) and administered Nano‐Glo substrate (Promega, United States) diluted 1:20 in PBS via subcutaneous injection (2.32 µL g^−1^ body weight; 75 µL mouse^−1^) (Figure 7D; Supplementary S2 Figure S9, Supporting Information).

### Statistical Analysis

The statistical analysis, including Student's t‐test, one‐way and two‐way analysis of variance (ANOVA), was performed using Prism GraphPad version 9.3.1 (GraphPad Software, Inc., La Jolla, CA, USA). Statistical significance was set as *p < 0.0332, **p < 0.0021, ***p < 0.0002, and ****p < 0.0001, with a 95% confidence interval.

## Conflict of Interest

The authors declare no conflict of interest.

## Author Contributions

N.S.P. and S.A. contributed equally to this work. A.S. supervised and directed the research. N.S.P. and S.A. wrote the manuscript and designed, performed, and analyzed all the manual and automated synthetic cells & CFPS in vitro and in vivo experiments; S.A. and O.K. developed the scripts for the liquid handler; O.K. calibrated the liquid handler's liquid classes; S.A. and I.G. designed the neural network solution; I.G. wrote the code, consulted about data curation and trained the network. I.G, G.C. and J.S. assisted in improving the manuscript. R.M assisted in the in‐vivo studies. J.S. assisted in funding obtaining.

## Supporting information



Supporting Information

Supporting Information

Supporting Information

Supporting Information

Supporting Information

## Data Availability

The data that support the findings of this study are available from the corresponding author upon request. The neural network code, raw training data, and running samples used in this study are openly available in github at https://github.com/IdoGalil/syn‐cells‐classification.
