# Supplementary material for: Scaling Up Synthetic Cell Production Using Robotics and Machine Learning Toward Therapeutic Applications
Source: Adv Biol (Weinh). 2025 Mar 31;9(5):2400671. doi: 10.1002/adbi.202400671 (PMC12078883; doi:10.1002/adbi.202400671)
Supplement: Supplementary file 1 — Supporting Information [file ADBI-9-2400671-s004.pdf]

ADVANCED BIOLOGY

Supporting Information

for *Adv. Biology*, DOI 10.1002/adbi.202400671

Scaling Up Synthetic Cell Production Using Robotics and Machine Learning Toward Therapeutic Applications

Noga Sharf-Pauker, Ido Galil, Omer Kfir, Gal Chen, Rotem Menachem, Jeny Shklover, Avi Schroeder and Shanny Ackerman**

Protein-Producing Synthetic Cells Production in High Volumes

Cell Free Protein Synthesis (CFPS) Inner and Feeding Solution: 30 minutes

(1) CFPS Pre-Inner Solution:

***Based on Adir, O. et al. J Vis Exp 158 (2020) with minor adjustments**

1. Take out the stock solutions from the -20°C freezer, and let them thaw at room temperature.
2. Vortex each stock solution well before adding the proper amount to your pre-inner solution according to the following table: (the pre-inner solution volumes mentioned in the table are according to the final CFPS inner solution volume that can be prepared using this pre-inner solution)

The solution preparation can be performed using a liquid handler, as detailed in the paper, or manually.

Reagent	Stock conc.		Final conc.		100µl	5,000µl	15,000µl
HEPES KOH (pH=8)	1	M	55	mM	5.5 µl	275 µl	825 µl
Magnesium acetate	1	M	14	mM	1.4 µl	70 µl	210 µl
Potassium acetate	1	M	50	mM	5.0 µl	250 µl	750 µl
Ammonium acetate	5.2	M	155	mM	3.0 µl	149 µl	447 µl
Polyethylene glycol 6000*	50	%W/V	1.5	%W/V	3.0 µl	150 µl	450 µl
3-PGA	0.5	M	40	mM	8.0 µl	400 µl	1200 µl
Amino acids - mixture I**	50	mM	2.5	mM	5.0 µl	250 µl	750 µl
Amino acids - mixture II***	50	mM	2.5	mM	5.0 µl	250 µl	750 µl
ATP	100	mM	1.2	mM	1.2 µl	60 µl	180 µl
GTP	50	mM	1	mM	2.0 µl	100 µl	300 µl
UTP	100	mM	0.8	mM	0.8 µl	40 µl	120 µl
IPTG	100	mM	1	mM	1.0 µl	50 µl	150 µl
Sucrose	2	M	200	mM	10.0 µl	500 µl	1500 µl

* The Polyethylene glycol 6000 stock solution is very viscous. Make sure to use a dedicated pipette to take the proper amounts.

** Amino acids - mixture I- Hydrophilic amino acids- 50mM of each of the following amino acids: Alanine, Arginine (HCl), Asparagine, Aspartic acid, Cysteine, Glutamic acid, Glutamine, Glycine, Histidine (HCl), Isoleucine, Leucine, Lysine, Methionine, Threonine, Proline, Serine, Valine.

*** Amino acids - mixture II- Hydrophobic amino acids- 50mM of each of the following amino acids: Phenylalanine, Tryptophan, Tyrosine. Due to the hydrophobic properties of these amino acids, they do not dissolve. Mix this solution especially well before adding from it to the Mix solution.

3. Mix the Pre-inner solution well, divide it into aliquots, and freeze it in a -20°C freezer.

The solution can be kept frozen for a month.

(2) Feeding Solution:

***Based on Adir, O. et al. J Vis Exp 158 (2020) with minor adjustments**

In the same manner as the Inner Solution, thaw the stock solutions at room temperature and vortex them well before adding the proper amount to your feeding solution, according to the following table:

**Reagent**	**Stock conc.**		**Final conc.**		**100µl**	**5,000µl**	**15,000µl**
HEPES KOH (pH=8)	1	M	83.3	mM	8.3 µl	416.5 µl	1249.5 µl
Magnesium acetate	1	M	21.2	mM	2.1 µl	106 µl	318 µl
Potassium acetate	1	M	75.5	mM	7.6 µl	377.5 µl	1132.5 µl
Ammonium acetate	5.2	M	236.4	mM	4.5 µl	227.3 µl	682 µl
Polyethylene glycol 6000	50	%W/V	4.54	%W/V	9.0 µl	450 µl	1350 µl
3-PGA*	0.5	M	60.1	mM	12.0 µl	601 µl	1803 µl
Amino acids - mixture I	50	mM	3.8	mM	7.6 µl	380 µl	1140 µl
Amino acids - mixture II	50	mM	3.8	mM	7.6 µl	380 µl	1140 µl
ATP	100	mM	1.8	mM	1.8 µl	90 µl	270 µl
GTP	50	mM	1.5	mM	3.0 µl	150 µl	450 µl
UTP	100	mM	1.2	mM	1.2 µl	60 µl	180 µl
IPTG	100	mM	1.5	mM	1.5 µl	75 µl	225 µl
Glucose	2	M	200	mM	10 µl	500 µl	1500 µl
Ultra-pure water (UPW)					23.7 µl	1186.7 µl	3560 µl

* The 3-PGA solution can be replaced with additional UPW without significantly changing the synthetic cells' activity

Mix the Feeding solution well, divide it into aliquots, and freeze it in a -20°C freezer. The solution can be kept frozen for a month.

<u>Lipids in Mineral Oil Solution</u>: 1.5 hours

Materials:

1-palmitoyl-2-oleoyl-glycero-3-phosphocholine (POPC) powder

Cholesterol powder

Chloroform

Mineral oil

Preparation of 10ml of 20mg/ml lipid solutions:

1. Take out the lipid powders from the -20°C freezer, and let them warm to room temperature for 20 minutes. Warm a 1.5ml vial shaker to 80°C inside a chemical hood.
2. For each of the lipids separately, weigh 200mg of the lipid powder into 15ml vial.
3. In a chemical hood, add 2.5 mL of chloroform to each vial.
4. Vortex well, until all the powder dissolves.
5. In a chemical hood, add 10ml of mineral oil to each one of the vials.
6. Vortex well.
7. Divide the lipid solutions into aliquots, 1ml of one type of lipid solution in each 1.5ml vial.
8. Open the caps of the 1.5ml vials and put them to heat in the pre-heated 1.5ml vial shaker for 1hr, , 80°C, at 450rpm.
9. Close and let the 1.5ml vials cool to room temperature, and then freeze them in a -20°C freezer.

The solutions can be kept frozen for a month.

<u>Synthetic Cells Production Process:</u> 1.5 hours preparation

Materials:

Pre-inner solution (section 1)

Pre-prepared *E.coli* S30 lysate (according to Krinsky, N. et al. A Simple and Rapid Method for Preparing a Cell-Free Bacterial Lysate for Protein Synthesis. PLoS One 11 (2016) with minor modifications: the bacterial growth volume was increased to 1500 ml, S30 buffer was prepared without 2-mercaptoethanol, and was enriched with 2 mM DTT).

DNA plasmid solution with the desired protein gene under T7 RNA polymerase promoter (purified DNA plasmid stock concentration above 85 ng/µl)

UPW

Feeding solution (section 2)

POPC and cholesterol stock solutions (section 3)

200mM Glucose solution

Synthetic Cells Production:

1. Thaw the POPC and cholesterol solutions at 37°C for several minutes, thaw the DNA solution, the pre-inner and feeding solutions at room temperature, and thaw the *E.coli* S30 lysate on ice. Cool a 15ml vial centrifuge and a 1.5ml vial centrifuge to 4°C.
2. Vortex the lipid stock solutions well.
3. **Lipid mixture:** The final lipid solution volume is twice the volume of the desired CFPS inner solution final volume (1:2 v:v CFPS solution:lipid solution), with an internal v:v ratio of 1:1 between the lipids (POPC: Cholesterol). Add to a new vial the proper amounts and mix the final solution well.

For example, for 100µl of CFPS inner solution, mix 100µl of POPC solution with 100µl of cholesterol solution.
A fluorophore conjugated to a lipid may be added to the lipid solution to mark the synthetic cells' membrane. In this work Rhodamine-labeled phospholipid (14:0 Liss Rhod PE, 1 mg/ml in ethanol) (Avanti Lipids Polar, Alabaster, AL) was incorporated by adding 0.4 µL to every 100 µL of lipid solution in mineral oil.

4. **CFPS inner solution:** vortex the Pre-inner solution well, and mix gently by pipetting the DNA solution and the *E.coli* S30 lysate solutions. Add each in the proper amount to a new vial according to the following table:

**Reagent**	**Final conc.**		**100µl**	**5,000µl**	**15,000µl**
Pre-Inner solution	52.5	%	52.5 µl	2625 µl	7875 µl
DNA	10	ng/µl	x	x	x
UPW	Up to the final volume		13.5 µl - x	675 µl - x	2025 µl -x
E.coli S30 lysate*	34	%	34 µl	1700 µl	5100 µl

* Add the *E.coli* S30 lysate last, after mixing the solution well by pipetting

5. **Emulsification:**
Manual emulsification- up to 200µl CFPS inner solution:
a. With a 200µl pipette, take the CFPS inner solution and pipette it vigorously into the lipid solution for 30 seconds. Make sure to move the tip around all the lipid solution, in order to create a homogeneous solution.
b. Immediately after, vortex the resulting solution gently on a vortex (around half of the maximum intensity of your vortex, the intensity may need calibration for your specific vortex) for another 30 seconds.

Manual emulsification- 200µl-500µl CFPS inner solution:
a. Add the CFPS inner solution into the lipid solution.
b. Quickly, switch pipettes to a 200µl pipette and pipette the solution vigorously for 1 minute. Make sure to move the tip around the lipid solution, in order to create a homogeneous solution.
c. Vortex the resulting solution gently on a vortex (around half of the maximum intensity of your vortex, the intensity may need calibration for your specific vortex) for another 1 minute.

Manual emulsification- 500µl-1000µl CFPS inner solution:

d. Add the CFPS inner solution into the lipid solution.
e. Quickly, switch pipettes to a 200µl pipette and pipette the solution vigorously for 1.5 minute. Make sure to move the tip around the lipid solution, in order to create a homogeneous solution.
f. Vortex the resulting solution gently on a vortex (around half of the maximum intensity of your vortex, the intensity may need calibration for your specific vortex) for another 1 minute.

GentleMACS emulsification- above 500µl CFPS inner solution:

a. In the GentleMACS, prepare a program of 200RPM, 1 minute mixing.
b. Transfer the lipid solution to GentleMACS M-tubes (each vial can accommodate up to 10ml of total solution: up to 6ml of lipid mixture solution and 3ml of CFPS Inner solution per tube).
c. Add the appropriate amount of CFPS inner solution to each M-tube, mix the solution by briefly pipetting to evenly distribute the CFPS inner solution in the lipid mixture, and quickly transfer the tube to the GentleMACS for mixing.

6. Incubate the emulsion in ice for 10 minutes, for emulsion stabilization.
7. In the meanwhile, add 1.5ml-4.5ml of 200mM glucose solution to a 15ml vial (corresponding 500ul – 3ml CFPS inner solution).
8. Gently drip the emulsion to the 15ml vial, on top of the glucose solution.
9. Centrifuge for 10 minutes, 100g, 4°C and directly continue to 10 minutes, 400g, 4°C centrifugation.
10. **Pellet extraction**:
    a. With a pipette, remove as much of the top oil layer as possible.
    b. With a pipette with a 1ml tip that **its end was trimmed,** aspirate about 500µl-1000µl of 200mM glucose solution.
    c. Insert the tip to the solution in the 15ml vial, while dispensing the glucose solution from the tip (helps reduce the accumulation of oil on the tip's end). Be careful not to disturb the pellet while dispensing.
    d. Extract the pellet.
    e. Before transferring the pellet to a clean 1.5ml vial, gently wipe the outside of the pipette tip with a Kimwipe to minimize oil residue transferred with the synthetic cell pellet.
    f. Dispense the synthetic cells pellet solution in a single, quick motion to a new 1.5ml tube. Avoid pipetting. If necessary, repeat steps 10b-10e until all the pellet is extracted.
11. Centrifuge for 10 minutes, 1000g, 4°C.
12. Gently remove as much of the supernatant as possible.
13. Resuspend your synthetic cells pellet with the feeding solution, in the same volume as the original CFPS inner solution.
14. Pipette the solution well with a 200ul pipette tip to reduce synthetic cells aggregation.
15. Incubate your synthetic cells suspension at 37°C for protein expression, without shaking. For the production of sfGFP, firefly luciferase and nano-luciferase, incubate for 2 hours.
