# Supplementary material for: Scaling Up Synthetic Cell Production Using Robotics and Machine Learning Toward Therapeutic Applications
Source: Adv Biol (Weinh). 2025 Mar 31;9(5):2400671. doi: 10.1002/adbi.202400671 (PMC12078883; doi:10.1002/adbi.202400671)
Supplement: Supplementary file 2 — Supporting Information [file ADBI-9-2400671-s005.pdf]

ADVANCED BIOLOGY

Supporting Information

for *Adv. Biology*, DOI 10.1002/adbi.202400671

Scaling Up Synthetic Cell Production Using Robotics and Machine Learning Toward Therapeutic Applications

Noga Sharf-Pauker, Ido Galil, Omer Kfir, Gal Chen, Rotem Menachem, Jeny Shklover, Avi Schroeder and Shanny Ackerman**

Supplementary 2:

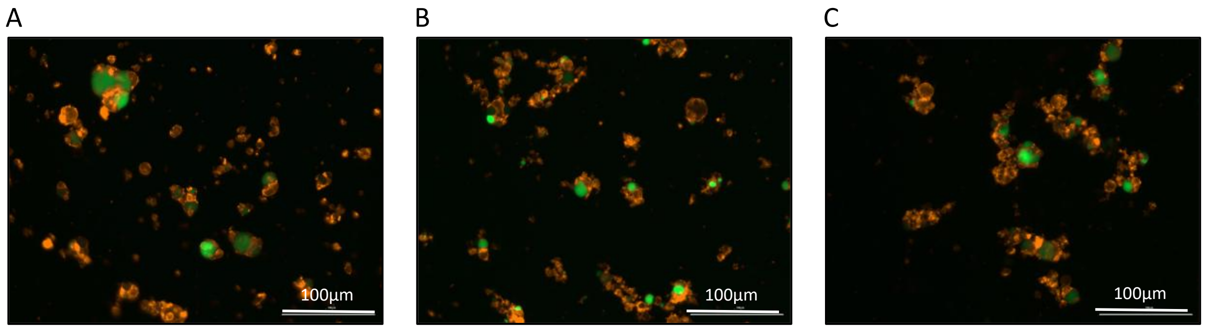

Figure S1: Representative images of sfGFP-producing-SCs prepared with frozen lipid mix solution. (A) SCs prepared with fresh lipid mix solution (B) SCs prepared with the same lipid mix solution after been stored for two weeks at -20◦c. (C) SCs prepared with the same lipid mix solution after been stored for a month at -20◦c. *Produced GFP (in green), Rhodamine –labeled membrane (in orange).* The images were taken using a fluorescent microscope with RFP and GFP filters, merged.

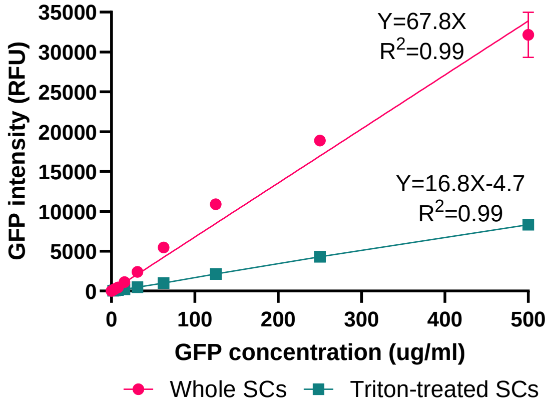

Figure S2: Calibration curve of GFP-purified protein mixed with either whole non-producing SCs (harboring the empty pET9a plasmid) or Triton-treated non-producing SCs, aimed at minimizing SC membrane absorbance effects and validating GFP intensity measurements. Fluorescence intensity values were measured using a plate reader (n = 3).

GFP concentration in SCs:

GFP stock concentration, according to Bradford assay = 9.4 mg/ml.

GFP concentration inside SCs (n=9):

- Whole sfGFP-producing-SCs: $\frac{(319.9 \pm 8.9)RFU}{67.8\frac{RFU}{\mu g/ml}} = (4.7 \pm 0.1)\frac{\mu g}{ml}$
- Triton-treated sfGFP-producing-SCs: $\frac{(74.3 \pm 3.1)RFU + 4.7RFU}{16.8\frac{RFU}{\mu g/ml}} = (4.7 \pm 0.5)\frac{\mu g}{ml}$

ImageStream SC GFP intensity (n=6):

$$(2.9 \pm 0.4) * 10^5 ImageStreamRFU = (4.7 \pm 0.5)\frac{\mu g}{ml}$$

$$(2.9 \pm 13.8\%) * 10^5 ImageStreamRFU = (4.7 \pm 10.6\%)\frac{\mu g}{ml}$$

$$10^5 ImageStreamRFU = (1.62 \pm 39.5\%)\frac{\mu g}{ml} = (1.62 \pm 0.64)\frac{\mu g}{ml}$$

GFP concentration in SCs produced by the original production process:

$$1.1 * 10^5 ImageStreamRFU = (1.78 \pm 0.70)\frac{\mu g}{ml}$$

GFP concentration in SCs produced by the optimized production process:

$$2.2 * 10^5 ImageStreamRFU = (3.56 \pm 1.41)\frac{\mu g}{ml}$$

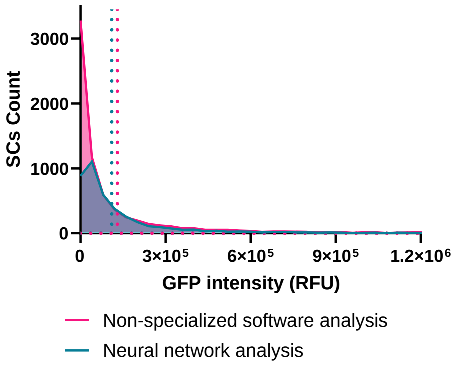

Figure S3: Comparison of protein-producing SCs population analysis differences between non-specialized software analysis and our neural network analysis of SCs produces by the original production process.

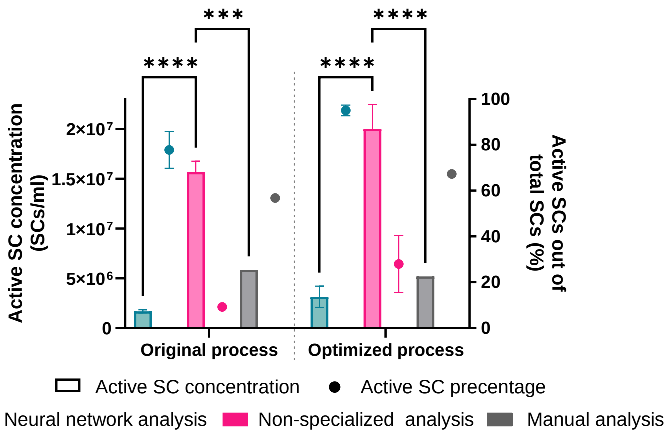

Figure S4: Classification differences of active SC concentration and percentage between neural network analysis, non-specialized analysis software and manual analysis of the same samples. For neural network and non-specialized software analyses, 10,000 particles per sample were sorted, whereas manual analysis used 1,000 particles per sample. Data are expressed as mean ± s.e.m. Two-way ANOVA with adjusted P value in Tukey multiple comparisons tests, ***P=<0.0002, ****P=<0.0001 (n = 3 independent samples).

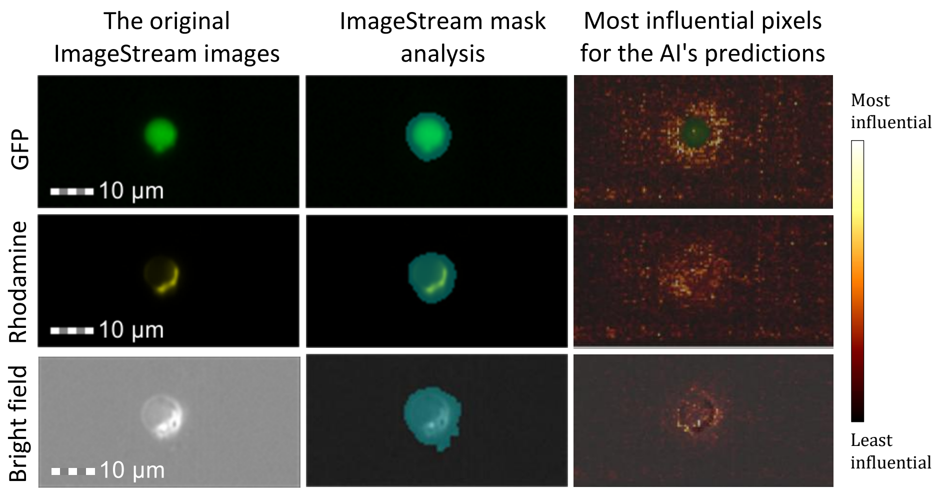

Figure S5: Illustrations of the IDEAS software analysis and the integrated gradients analysis of our neural network, both conducted on a representative image from imaging flow cytometry.

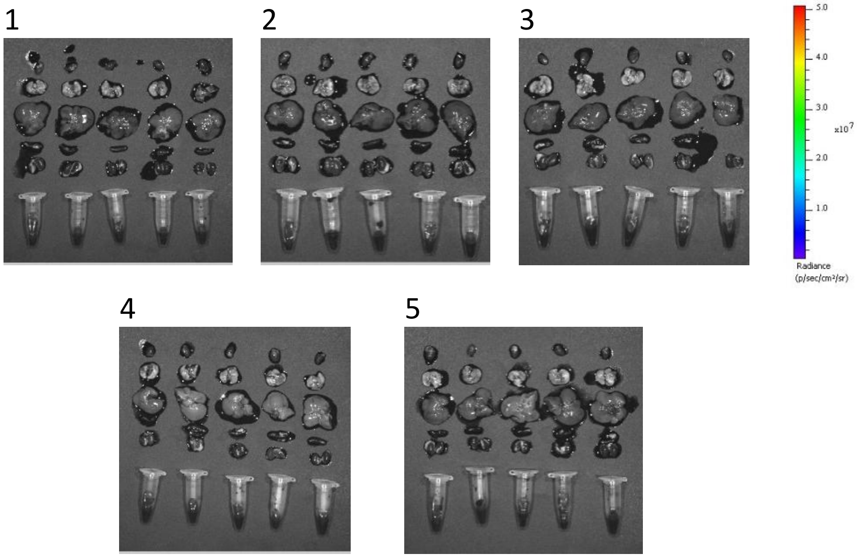

Figure S6: Biodistribution of Nano-luciferase (NanoLuc)-producing SCs in mouse organs over time, after subcutaneous injection (150 µl/mouse) into C57 mice. (Left: PBS; middle to right: SCs; top to bottom: heart, lungs, liver, spleen, kidneys, and blood). The luminescent signal from the particles in the organs was monitored using IVIS imaging at 0, 1, 2, 5, and 24 hours after SC administration in (1)-(5), respectively. Images were taken 25 minutes following substrate administration (Nano-Glo substrate, diluted 1:20 in PBS) via both IP and subcutaneous injections (4.65 µl/g, total 150 µl/mouse).

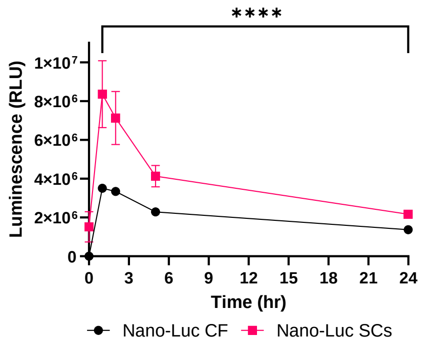

Figure S7: Nano-Luc-producing CFPS solution and Nano-Luc-producing SCs activity overtime. Data are expressed as mean ± s.e.m (*n* = 3 independent samples). Two-way ANOVA with adjusted P value in Tukey multiple comparisons tests, ****P=<0.0001.

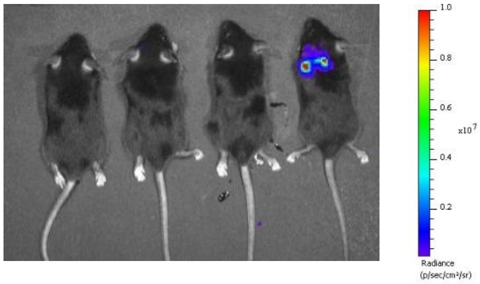

Figure S8: NanoLuc producing SCs luminescence. C57 mice were injected subcutaneously (150 ul/mouse of SCs or PBS, Left to right – PBS, Nanoluc producing SCs without substrate, SCs without DNA template with substrate, Nanoluc producing SCs with substrate). The luminescent signal from the particles was monitored using whole-animal imaging. Images were taken 5 minutes following substrate administration (Nano-Glo substrate, diluted 1:20 in PBS) via subcutaneous injection (total 100ul/mouse).

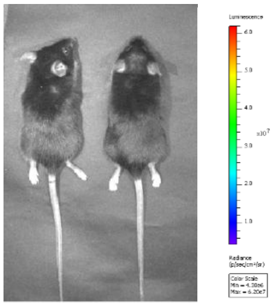

Figure S9: PBS-injected mice were used as controls to compare variability in SC samples produced via automated or manual production methods in vivo (100 µL/mouse).

Table S1: Examples of simple-to-classify **active** SCs:
A transparent bubble can be detected in the Brightfield channel, encapsulated within a Rhodamine-labeled membrane and filled with detectable GFP signal.

**Brightfield channel**	**Rhodamine channel**	**GFP channel**
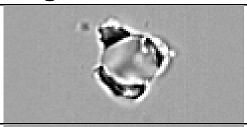	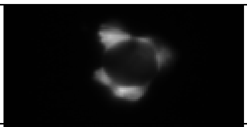	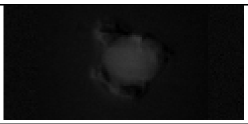
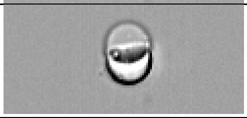	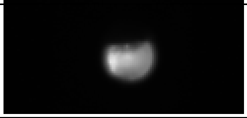	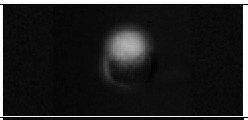
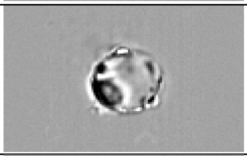	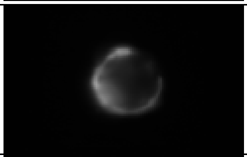	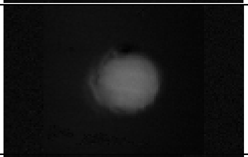
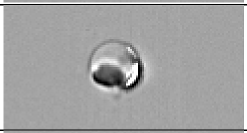	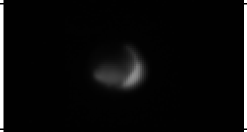	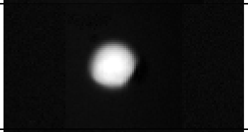
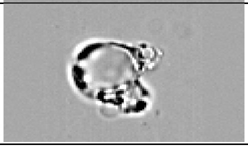	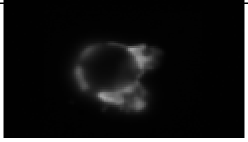	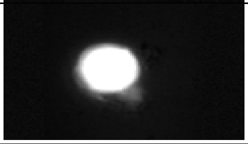

Table S2: Examples of simple-to-classify **inactive** SCs:
A transparent bubble can be detected in the Brightfield channel, encapsulated within a Rhodamine-labeled membrane and empty of GFP signal. Occasionally, autofluorescence from the Rhodamine-labeled membrane can be detected in the GFP channel, colocalizing with the Rhodamine signal.

**Brightfield channel**	**Rhodamine channel**	**GFP channel**
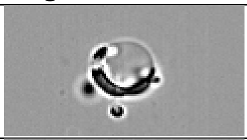	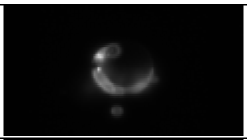	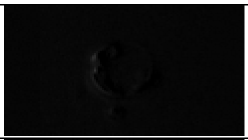
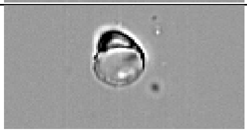	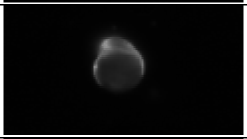	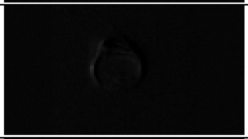
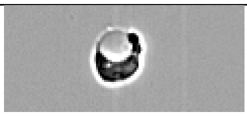	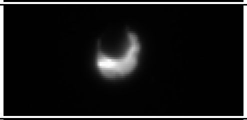	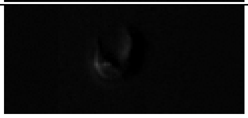
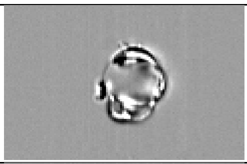	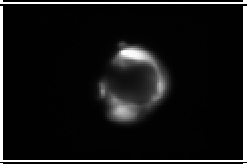	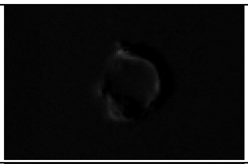
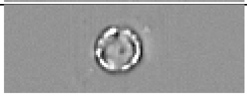	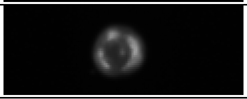	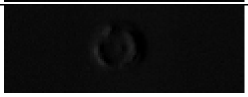

Table S3: Examples of the visual diversity of **active** SCs:

Brightfield channel	Rhodamine channel	GFP channel
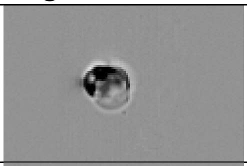	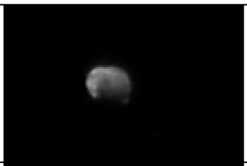	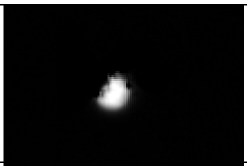
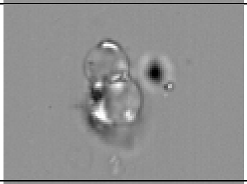	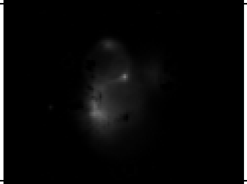	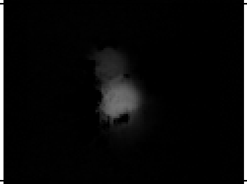
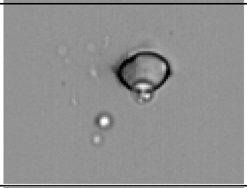	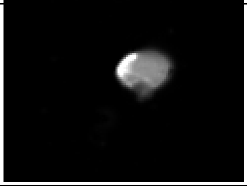	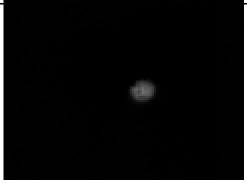
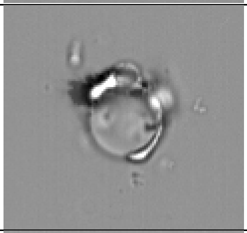	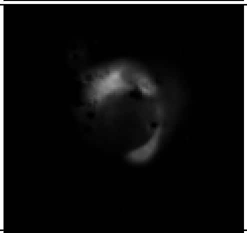	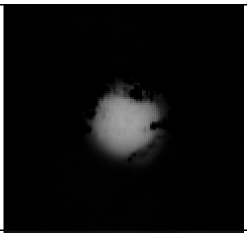
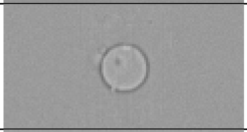	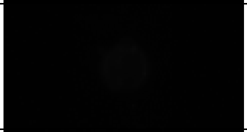	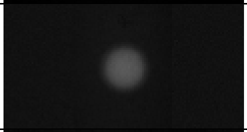
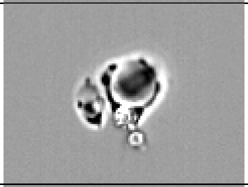	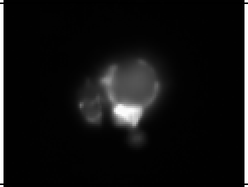	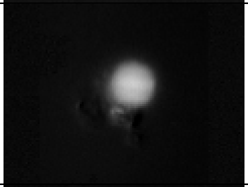
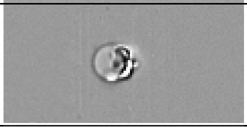	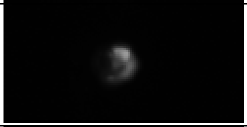	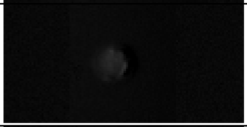
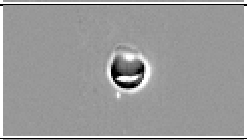	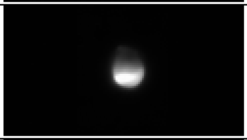	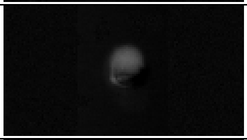
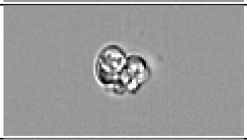	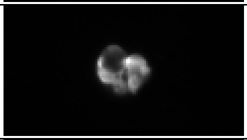	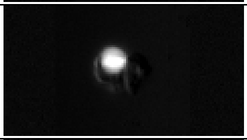
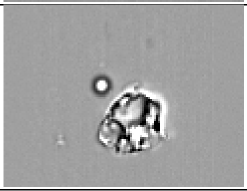	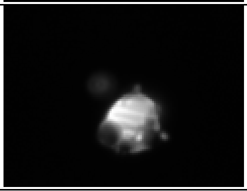	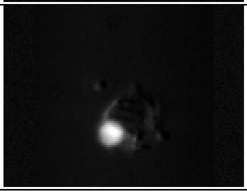

Table S4: Examples of the visual diversity of **inactive** SCs:

**Brightfield channel**	**Rhodamine channel**	**GFP channel**
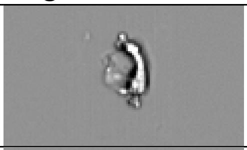	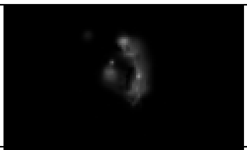	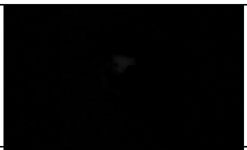
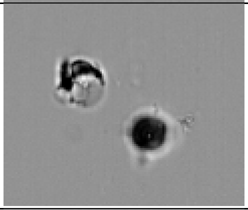	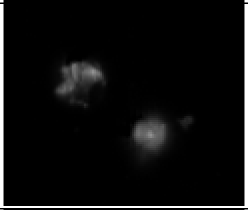	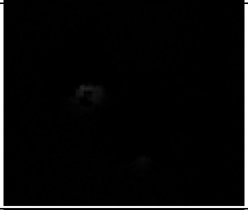
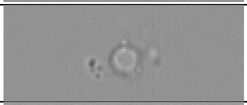	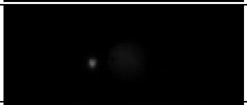	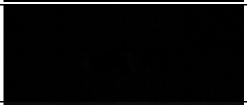
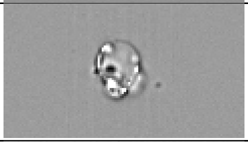	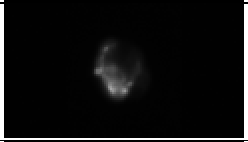	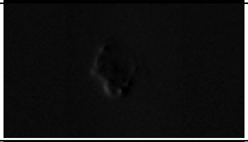
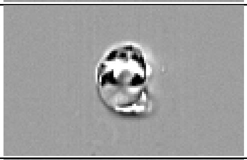	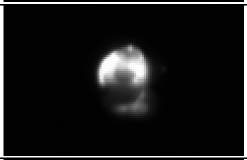	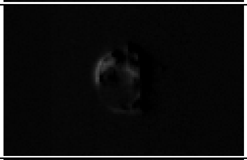
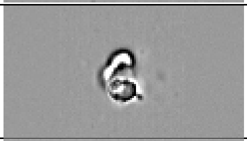	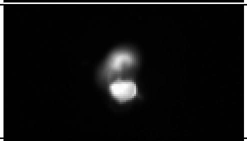	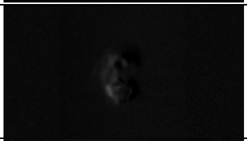
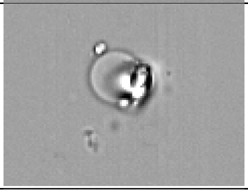	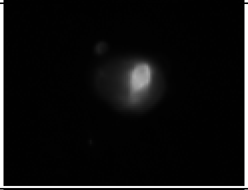	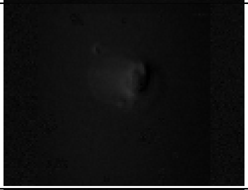
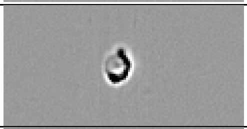	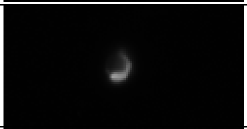	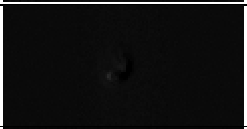
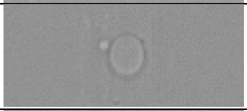	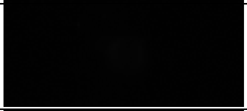	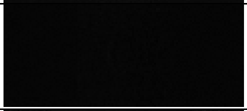
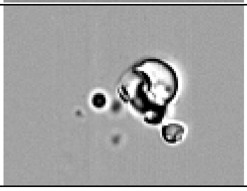	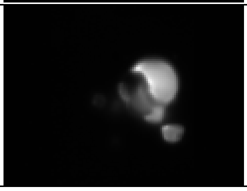	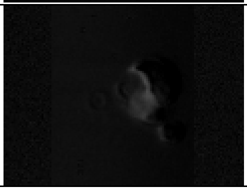

Table S5: Examples of the visual diversity of **oil droplets**:

Structures lacking the defining features of SCs.

**Brightfield channel**	**Rhodamine channel**	**GFP channel**
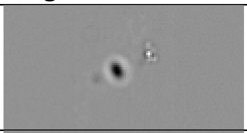	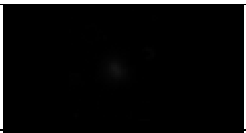	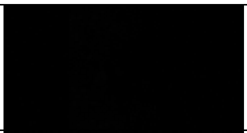
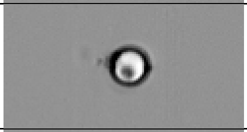	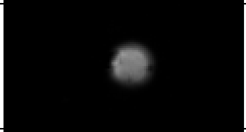	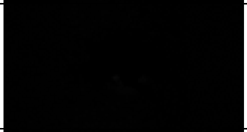
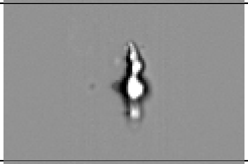	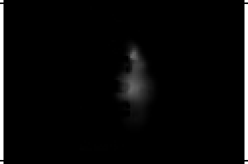	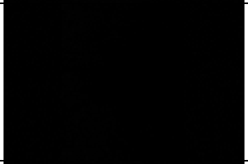
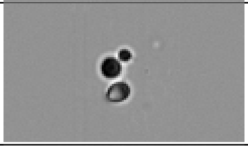	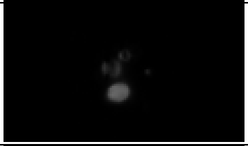	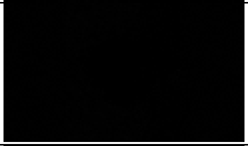
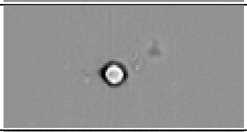	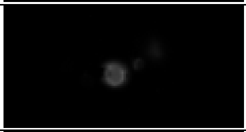	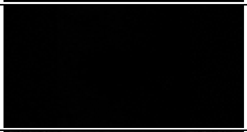
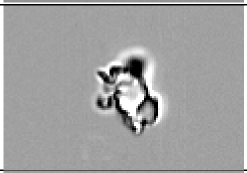	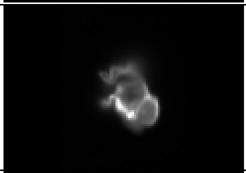	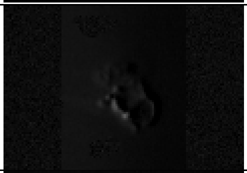
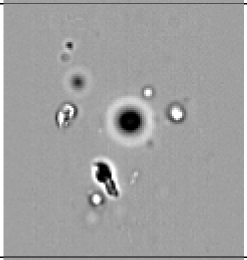	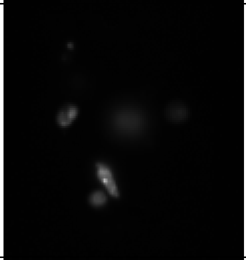	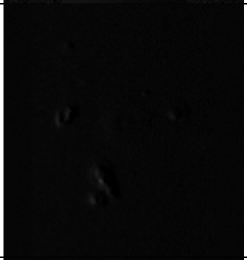
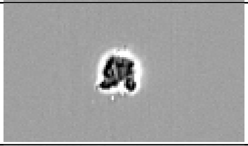	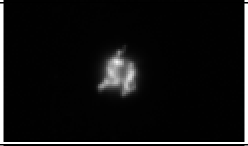	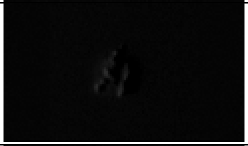
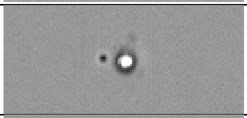	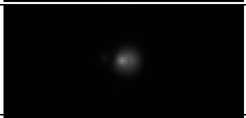	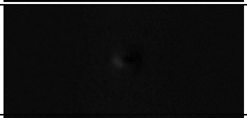
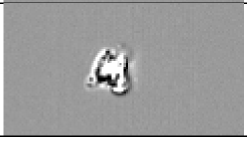	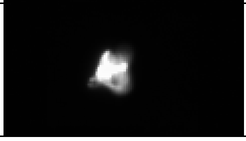	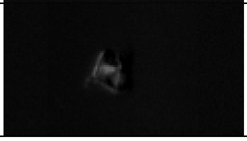

Materials and Methods:

1. *E.coli* S30-T7 Lysate preparation:

E.coli S30 bacterial lysate was prepared as previously described[1,2] from BL21(DE3) E. coli (New England Biolabs, USA) transformed with the T7 polymerase-expressing TargeTron vector pAR1219 (Sigma-Aldrich, Rehovot, Israel), with minor modifications: the bacterial growth volume was increased to 1500 ml, S30 buffer was prepared without 2-mercaptoethanol, and was enriched with 2 mM DTT instead of 1 mM DTT.

2. SCs inner reaction composition based on *E.coli* S30-T7 lysate and the corresponding Feeding solution.

The CFPS inner solution, and the feeding solution were prepared as previously described[1,2] with minor modification: the final concentration of PEG6000 was reduced by half from 3% to 1.5% w/v for the inner solution, and from 4.5% to 2.25% w/v for the Feeding solution (Table S6).

Table S6: Optimal CFPS inner and feeding solutions composition for in-house *E.coli*-based SCs

Number	Reagent	Stock concentration	Inner solution - final concentration	Feeding solution - final concentration
1	HEPES KOH pH=8	1 M	55 mM	83 mM
2	Magnesium acetate	1 M	14 mM	21 mM
3	Potassium acetate	1 M	50 mM	76 mM
4	Ammonium acetate	5.2 M	155 mM	236.4 mM
5	Polyethylene glycol (PEG) 6000	50% w/v	1.5% w/v	2.25% w/v
6	3-Phosphoglyceric acid (3-PGA)	0.5 M	40 mM	61 mM
7	Amino acids - mixture I	50 M	2.5 mM	3.8 mM
8	Amino acids - mixture II	50 M	2.5 mM	3.8 mM
9	ATP	100 mM	1.2 mM	1.8 mM
10	GTP	50 mM	1 mM	1.5 mM
11	UTP	100 mM	0.8 mM	1.2 mM
12	IPTG	100 mM	1 mM	1.5 mM
13	Sucrose	2 M	200 mM	-
14	Glucose	2 M	-	303 mM
15	S30-T7 lysate		34% v/v	-
16 **	DNA plasmid		10 ng/µL	-
17 *	Ultrapure water (UPW)		To total reaction volume	18.3 % (v/v)
** plasmid DNA volume addition according to stock concentration				
* UPW should be added to complete the final required volume				

3. DNA vectors:

The DNA template encoding super-folder GFP (sfGFP) was procured from Sandia BioTech (Albuquerque, New Mexico, USA). This DNA template was then cloned into a pET9a vector, incorporating a histidine×6 tag, as previously described in a study conducted by our group[1].

A Nano-Luciferase (NanoLuc) coding plasmid was purchased from Addgene (pTXTL-T7max-NanoLuc plasmid #188313).

4. Protein sequences

Name	Sequence
sf-GFP-6His	CATATGAGCAAAGGAGAAGAACTTTTCACTGGAGTTGTCCCAATTCTTGTTGAATTAGATGGTG<br>ATGTTAATGGGCACAAATTTTCTGTCCGTGGAGAGGGTGAAGGTGATGCTACAAACGGAAAAC<br>TCACCCTTAAATTTATTTGCACTACTGGAAAACTACCTGTTCCATGGCCAACACTTGTCACTACTC<br>TGACCTATGGTGTTCAATGCTTTTCCCGTTATCCGGATCACATGAAACGGCATGACTTTTTCAAG<br>AGTGCCATGCCCGAAGGTTATGTACAGGAACGCACTATATCTTTCAAAGATGACGGGACCTAC<br>AAGACGCGTGCTGAAGTCAAGTTTGAAGGTGATACCCTTGTTAATCGTATCGAGTTAAAAGGT<br>ATTGATTTTAAAGAAGATGGAAACATTCTCGGACACAAACTCGAGTACAACTTTAACTCACACA<br>ATGTATACATCACGGCAGACAAACAAAAGAATGGAATCAAAGCTAACTTCAAAATTCGCCACA<br>ACGTTGAAGATGGTTCCGTTCAACTAGCAGACCATTATCAACAAAATACTCCAATTGGCGATGG<br>CCCTGTCCTTTTACCAGACAACCATTACCTGTCGACACAATCTGTCCTTTCGAAAGATCCCAACG<br>AAAAGCGTGACCACATGGTCCTTCTTGAGTTTGTAACTGCTGCTGGGATTACACATGGCATGGA<br>TGAGCTCTACAAAGGAGGGTCCCATCACCATCACCATCACTAAGGATCC
NanoLuc	ATGGTCTTCACACTCGAAGATTTCGTTGGGGACTGGCGACAGACAGCCGGCTACAACCTGGACC<br>AAGTCCTTGAACAGGGAGGTGTGTCCAGTTTGTTTCAGAATCTCGGGGTGTCCGTAACTCCGATC<br>CAAAGGATTGTCCTGAGCGGTGAAAATGGGCTGAAGATCGACATCCATGTCATCATCCCGTATGA<br>AGGTCTGAGCGGCGACCAAATGGGCCAGATCGAAAAAATTTTTAAGGTGGTGTACCCTGTGGAT<br>GATCATCACTTTAAGGTGATCCTGCACTATGGCACACTGGTAATCGACGGGGTTACGCCGAACAT<br>GATCGACTATTTCGGACGGCCGTATGAAGGCATCGCCGTGTTCGACGGCAAAAAGATCACTGTAA<br>CAGGGACCCTGTGGAACGGCAACAAAATTATCGACGAGCGCCTGATCAACCCCGACGGCTCCCT<br>GCTGTTCCGAGTAACCATCAACGGAGTGACCGGCTGGCGGCTGTGCGAACGCATTCTGGCG

5. SCs' Outer solution composition

For 100 µL, 500 µL, 1 mL, and 3 mL of CFPS inner solution, volumes of 1.5 mL, 1.5 mL, 3 mL, and 4.5 mL, of 200 mM glucose dissolved in UPW, respectively, were prepared in 15 mL plastic vials.

6. SCs' Lipids

1-palmitoyl-2-oleoyl-sn-glycero-3-phosphocholine (POPC) was purchased from Lipoid (Ludwigshafen, Germany). Cholesterol was purchased from Sigma-Aldrich (Rehovot, Israel). Rhodamine-labeled phospholipid — 1,2-dimyristoyl-sn-glycero-3-phosphoethanolamine-N-(lissamine rhodamine B sulfonyl) (ammonium salt) (14:0 Liss Rhod PE) was purchased from (Avanti Lipids Polar, Alabaster, AL).

7. sfGFP In-Vitro Production in CFPS reaction

To assess the activity of the cell-free (CF) reaction, sfGFP fluorescence signal (measured at 488/530 nm) of samples prepared with in-house lysate-based were measured using an Infinite 200 PRO multimode reader (TECAN, Austria) controlled by the i-control 1.10 software.

8. Luminescence assays

Luminescence was measured using the Infinite 200PRO multimode reader (TECAN, Austria), controlled by the i-control 1.10 software. Immediately before measurement, the CFPS or SCs reactions were mixed in a 1:1 ratio with Nano-Glo substrate (Promega, United States) diluted 1:200.

9. Fluorescent Microscopy Analysis of SCs

The Lionheart FX automated microscope (BioTek, Santa Clara, CA), controlled by Agilent Gen5 software (version 3.11), was used to visualize and analyze sfGFP or NanoLuc-producing SCs, both were membrane-labeled with Rhodamine. The fabricated SCs were evaluated as follows: 5 µL of the SCs solution, either pre or post protein expression, was placed on a BSA-covered slide, and a cover slide was placed on top. For GFP-producing SCs, acquisition was performed using GFP and RFP filter cubes (EX 469/35 EM 525/39 and EX 531/40 EM 593/40, respectively) and LED cubes (465 nm and 523 nm, respectively), alongside brightfield visualization using 20X and 40X objectives. For NanoLuc-producing SCs, acquisition was performed using only the RFP filter cube (EX 531/40 EM 593/40) and LED cube (523 nm), alongside brightfield visualization using 20X and 40X objectives.

References:

1 Krinsky, N. *et al.* A simple and rapid method for preparing a cell-free bacterial lysate for protein synthesis. *PLoS One* **11**, e0165137 (2016).

2 Adir, O. *et al.* Preparing protein producing SCs using cell free bacterial extracts, liposomes and emulsion transfer. *JoVE (Journal of Visualized Experiments)*, e60829 (2020).
