# Supplementary material for: Scaling Up Synthetic Cell Production Using Robotics and Machine Learning Toward Therapeutic Applications
Source: Adv Biol (Weinh). 2025 Mar 31;9(5):2400671. doi: 10.1002/adbi.202400671 (PMC12078883; doi:10.1002/adbi.202400671)
Supplement: Supplementary file 3 — Supporting Information [file ADBI-9-2400671-s002.pdf]

ADVANCED BIOLOGY

Supporting Information

for *Adv. Biology*, DOI 10.1002/adbi.202400671

Scaling Up Synthetic Cell Production Using Robotics and Machine Learning Toward Therapeutic Applications

Noga Sharf-Pauker, Ido Galil, Omer Kfir, Gal Chen, Rotem Menachem, Jeny Shklover, Avi Schroeder and Shanny Ackerman**

Supplementary 3- Liquid handler (LiHa) properties and scripts

The employed automated LiHa station is a Tecan Freedom Evo 75 robot, a compact liquid handling platform for small life science laboratories. The device is equipped with a liquid handling arm (LiHa) with one channel for disposable tips (DiTis). The station is controlled by Freedom EVOware v2.8 (Tecan Group Ltd.). The software interface, together with the LiHa's layout, is depicted here in Supplementary 3, Figure S1.

LiHa calibration is essential for optimal performance and requires careful adjustments for each type of liquid being handled. The TECAN EVOware standard V2.8 software includes predefined Liquid Classes with properties suitable for various liquids. However, customization is recommended for non-standard solutions, allowing users to edit Liquid Class parameters and personalize pipetting settings for each liquid. To effectively calibrate the LiHa for a new type of liquid, it is essential to know its density, viscosity, and electrical conductivity in advance. These properties significantly influence handling characteristics and are critical for making accurate adjustments. With approximately 25 parameters available for adjustment, such as aspirate and dispense rates, air gaps, and tip type, precision is prioritized. In the LiHa workstation, pipetting parameters (e.g., aspiration speed, air gap) mainly affect precision, while calibration parameters (e.g., slope and offset of the calibration curve) are essential for accuracy[1]. An illustrative overview of LiHa's conceptual calibration process per specific liquid type is provided in Figure 3. While most solutions used in the Cell Free Protein Synthesis (CFPS) pre-inner and feeding solutions required only minor adjustments to the 'PBS Liquid Class' for accurate handling, solutions such as PEG 6000, sucrose, and lipid mixtures necessitated precise calibration to ensure accuracy. In this work, the LiHa was utilized to assemble solutions necessary for SC production, including the CFPS pre-inner, lipid mixture, and feeding solutions. These solutions were prepared using stock solutions categorized into five liquid classes: hydrophilic solute solution, 50% w/w PEG, sucrose, ultrapure water (UPW), and mineral oil, with each class calibrated separately. For the hydrophilic solutions, several fluid sub-range volumes were tested and calibrated to ensure accurate and precise volume aspiration and dispensing. The liquid class parameters and pipetting conditions for each liquid class are presented here in Supplementary 3, Table S1. The complete scripts for preparing the CFPS pre-inner, lipid mixture and feeding solutions are available here in Supplementary 3, Scripts 1 and 2. Additionally, the script for the emulsification step using the LiHa can be found in Supplementary 3, Script 3

Reference:

1 Bessemans, L. *et al.* Automated Gravimetric Calibration to Optimize the Accuracy and Precision of TECAN Freedom EVO Liquid Handler. *Journal of Laboratory Automation* **21**, 693-705 (2016). https://doi.org:10.1177/2211068216632349

Table S1: LiHa parameters for each Liquid Class

	parameters	Hydrophilic solutions									PEG-6000 50 $\frac{w}{w}$%	Sucrose	UPW	Mineral oil
Target Volume (μl)		12	15	18	21	30	45	75	82.5	120	90	150	180	200
Measured Volume (μl)		12.07 ± 0.08	15.17 ± 0.72	18.10 ± 0.13	20.87 ± 0.18	29.97 ± 0.07	45.30 ± 0.11	75.62 ± 0.17	83.00 ± 0.14	121.25 ± 0.23	91.40 ± 0.20	151.67 ± 0.26	181.30 ± 0.31	201.95 ± 0.23
Global type	Liquid conductivity	good									good			bad
Aspiration parameters	Aspiration speed($\frac{\mu l}{sec}$)	10						100			10	10	100	10
	Delay (ms)	500									9000	1000	300	9000
	STAG (μl)	19						20			20	200	20	20
	LAG (μl)	1									8	10	5	15
	TAG (μl)	0						1			2	1	10	10
	Excess volume (μl)	0												
	Conditioning Volume (μl)	0												
	Aspiration position	Liquid level + 2 mm offset, with tracking									Liquid level + 2 mm offset, with tracking			Z-max + -0.5 mm offset, no tracking
	Retract tips to	-5 mm from Liquid level									-5 mm from Liquid level			Z-start + -5 mm offset, no tracking
	Retract speed($\frac{mm}{sec}$)	20									10			8
Dispense parameters	Dispense speed($\frac{\mu l}{sec}$)	50						100			15	20	200	15
	Break-off speed ($\frac{\mu l}{sec}$)	37.5						75			10	15	150	10
	Delay (ms)	1000									1000	2000	0	10000

	Dispense position	Z-max + -0.5 mm offset, no tracking									Liquid level + 1 mm offset, with tracking	Liquid level + 2 mm offset, with tracking	Liquid level + 1 mm offset, with tracking	Z-dispense + 2 mm offset, no tracking
	Tip touching	-									-			Moves tips to front, speed 10 $\frac{mm}{s}$, 1000 ms delay after touching
	Retract tips to	z-dispense									z-dispense			z-travel
	Retract speed($\frac{mm}{sec}$)	10									5	10	10	3
Calibration parameters	Offset (μl)	0.95	0.5	0.96	0.58	1.38	1. 5	2.3	1.1	1.28	0.4	0.9	1.13	2.9
	Factor	1.0905	1.0750	1.0650	1.0620	1.0442	1.0352	1.0221	1.0164	0.9500	0.9300	1.032	1.009	1.016

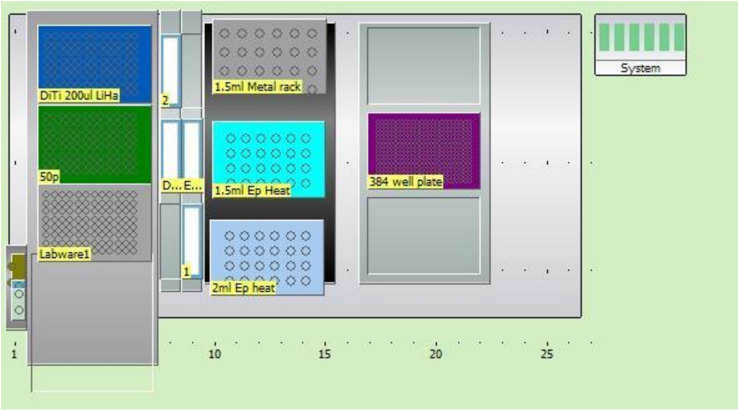

Figure S1: Freedom EVOware v2.8 user interface and schematic layout of the LiHa station.

Script 1- CFPS pre-inner solution preparation script for a final volume of 1500µl CFPS solution:

```
Group("Initialization");

UserPrompt("check all the eppendorfs are open",0,-1);

GroupEnd();

Group("Initial Wash");

Wash(1,1,0,1,2,"30.0",500,"4.0",500,10,70,30,1,0,1000,0);

Wash(1,1,0,1,2,"5.0",500,"4.0",500,10,70,30,0,0,1000,0);

GroupEnd();

Vector("Move Roma","17","2",0,1,2,2,0,0);

Group("round 1");

BeginLoop("1","3 rounds");

Group("Add HEPES KOH PH=8");

GetDITI2(1,"DiTi 200ul LiHa",0,0,10,70);

Mix(1,"CF wet contact","50",0,0,0,0,0,0,0,0,0,0,0,10,1,1,"06041000",10,0,0);

Aspirate(1,"CF wet contact","82.5",0,0,0,0,0,0,0,0,0,0,0,10,1,1,"06041000",0,0);

Dispense(1,"CF wet contact","82.5",0,0,0,0,0,0,0,0,0,0,0,10,2,1,"06041000",1,"3 rounds",0,1,0);

DropDITI(1,2,3,0,0,0);

GroupEnd();

Group("Initial Wash");

Wash(1,1,0,1,2,"10",500,"4.0",500,10,70,30,1,0,1000,0);

Wash(1,1,0,1,2,"5.0",500,"4.0",500,10,70,30,0,0,1000,0);

GroupEnd();
```

```
Group("Add magnesium acetate");

GetDITI2(1,"DiTi 50ul LiHa",0,0,10,70);

Mix(1,"CF wet contact","50",0,0,0,0,0,0,0,0,0,0,0,10,1,1,"0604@000",10,0,0);

Aspirate(1,"CF wet contact","21",0,0,0,0,0,0,0,0,0,0,0,10,1,1,"0604@000",0,0);

Dispense(1,"CF wet contact","21",0,0,0,0,0,0,0,0,0,0,0,10,2,1,"06041000",1,"3 rounds",0,1,0);

DropDITI(1,2,3,10,70,0);

GroupEnd();

Group("Initial Wash");

Wash(1,1,0,1,2,"10",500,"4.0",500,10,70,30,1,0,1000,0);

Wash(1,1,0,1,2,"5.0",500,"4.0",500,10,70,30,0,0,1000,0);

GroupEnd();

Group("Add potassium acetate");

GetDITI2(1,"DiTi 200ul LiHa",0,0,10,70);

Mix(1,"CF wet contact","75",0,0,0,0,0,0,0,0,0,0,0,10,1,1,"06040200",10,0,0);

Aspirate(1,"CF wet contact","75",0,0,0,0,0,0,0,0,0,0,0,10,1,1,"06040200",0,0);

Dispense(1,"CF wet contact","75",0,0,0,0,0,0,0,0,0,0,0,10,2,1,"06041000",1,"3 rounds",0,1,0);

DropDITI(1,2,3,10,70,0);

GroupEnd();

Group("Initial Wash");

Wash(1,1,0,1,2,"10",500,"4.0",500,10,70,30,1,0,1000,0);

Wash(1,1,0,1,2,"5.0",500,"4.0",500,10,70,30,0,0,1000,0);

GroupEnd();
```

```
Group("Add ammonium acetate");

GetDITI2(1,"DiTi 200ul LiHa",0,0,10,70);

Mix(1,"CF wet contact","50",0,0,0,0,0,0,0,0,0,0,0,0,10,1,1,"06040P00",10,0,0);

Aspirate(1,"CF wet contact","45",0,0,0,0,0,0,0,0,0,0,0,0,10,1,1,"06040P00",0,0);

Dispense(1,"CF wet contact","45",0,0,0,0,0,0,0,0,0,0,0,0,10,2,1,"06041000",1,"3 rounds",0,1,0);

DropDITI(1,2,3,10,70,0);

GroupEnd();

Group("Initial Wash");

Wash(1,1,0,1,2,"10",500,"4.0",500,10,70,30,1,0,1000,0);

Wash(1,1,0,1,2,"5.0",500,"4.0",500,10,70,30,0,0,1000,0);

GroupEnd();

Group("Add PEG 6000 *1.5% final conc*");

GetDITI2(1,"DiTi 200ul LiHa",0,0,10,70);

Mix(1,"PEG dispense for SC 50% w/w","50",0,0,0,0,0,0,0,0,0,0,0,0,10,1,1,"06040040",6,0,0);

Aspirate(1,"PEG dispense for SC 50% w/w","45",0,0,0,0,0,0,0,0,0,0,0,0,10,1,1,"06040040",0,0);

Dispense(1,"PEG dispense for SC 50% w/w","45",0,0,0,0,0,0,0,0,0,0,0,0,10,2,1,"06041000",1,"3 rounds",0,1,0);

Mix(1,"PEG dispense for SC 50% w/w","100",0,0,0,0,0,0,0,0,0,0,0,0,10,2,1,"06041000",3,1,"3 rounds",0,1,0);

DropDITI(1,2,3,10,70,0);

GroupEnd();

Group("Initial Wash");

Wash(1,1,0,1,2,"10",500,"4.0",500,10,70,30,1,0,1000,0);

Wash(1,1,0,1,2,"5.0",500,"4.0",500,10,70,30,0,0,1000,0);
```

```
GroupEnd();
Group("Add 3PGA 0.5M");
GetDITI2(1,"DiTi 200ul LiHa",0,0,10,70);
Mix(1,"CF wet contact","50",0,0,0,0,0,0,0,0,0,0,0,0,10,1,1,"060400p0",10,0,0);
Aspirate(1,"CF wet contact","120",0,0,0,0,0,0,0,0,0,0,0,0,10,1,1,"060400p0",0,0);
Dispense(1,"CF wet contact","120",0,0,0,0,0,0,0,0,0,0,0,0,10,2,1,"06041000",1,"3 rounds",0,1,0);
DropDITI(1,2,3,10,70,0);
GroupEnd();
Group("Initial Wash");
Wash(1,1,0,1,2,"10",500,"4.0",500,10,70,30,1,0,1000,0);
Wash(1,1,0,1,2,"5.0",500,"4.0",500,10,70,30,0,0,1000,0);
GroupEnd();
Group("Add AA1");
GetDITI2(1,"DiTi 200ul LiHa",0,0,10,70);
Mix(1,"CF wet contact","50",0,0,0,0,0,0,0,0,0,0,0,0,10,1,1,"06042000",10,0,0);
Aspirate(1,"CF wet contact","75",0,0,0,0,0,0,0,0,0,0,0,0,10,1,1,"06042000",0,0);
Dispense(1,"CF wet contact","75",0,0,0,0,0,0,0,0,0,0,0,0,10,2,1,"06041000",1,"3 rounds",0,1,0);
DropDITI(1,2,3,10,70,0);
GroupEnd();
Group("Initial Wash");
Wash(1,1,0,1,2,"10",500,"4.0",500,10,70,30,1,0,1000,0);
Wash(1,1,0,1,2,"5.0",500,"4.0",500,10,70,30,0,0,1000,0);
```

```
GroupEnd();
Group("Add AA2");
GetDITI2(1,"DiTi 200ul LiHa",0,0,10,70);
Mix(1,">> CF wet contact <<  924","50",0,0,0,0,0,0,0,0,0,0,0,10,1,1,"0604P000",15,0,0);
Aspirate(1,">> CF wet contact <<	920","75",0,0,0,0,0,0,0,0,0,0,0,10,1,1,"0604P000",0,0);
Dispense(1,">> CF wet contact <<	922","75",0,0,0,0,0,0,0,0,0,0,0,10,2,1,"06041000",0,0);
DropDITI(1,2,3,10,70,0);
GroupEnd();
Group("Initial Wash");
Wash(1,1,0,1,2,"10",500,"4.0",500,10,70,30,1,0,1000,0);
Wash(1,1,0,1,2,"5.0",500,"4.0",500,10,70,30,0,0,1000,0);
GroupEnd();
Group("Add ATP");
GetDITI2(1,"DiTi 50ul LiHa",0,0,10,70);
Mix(1,"CF wet contact","18",0,0,0,0,0,0,0,0,0,0,0,10,1,1,"06040400",10,0,0);
Aspirate(1,"CF wet contact","18",0,0,0,0,0,0,0,0,0,0,0,10,1,1,"06040400",0,0);
Dispense(1,"CF wet contact","18",0,0,0,0,0,0,0,0,0,0,0,10,2,1,"06041000",0,0);
DropDITI(1,2,3,10,70,0);
GroupEnd();
Group("Initial Wash");
Wash(1,1,0,1,2,"10",500,"4.0",500,10,70,30,1,0,1000,0);
Wash(1,1,0,1,2,"5.0",500,"4.0",500,10,70,30,0,0,1000,0);
```

```
GroupEnd();

Group("Add GTP");

GetDITI2(1,"DiTi 200ul LiHa",0,0,10,70);

Mix(1,"CF wet contact","50",0,0,0,0,0,0,0,0,0,0,0,0,10,1,1,"06040p00",10,0,0);

Aspirate(1,"CF wet contact","30",0,0,0,0,0,0,0,0,0,0,0,0,10,1,1,"06040p00",0,0);

Dispense(1,"CF wet contact","30",0,0,0,0,0,0,0,0,0,0,0,0,10,2,1,"06041000",1,"3 rounds",0,1,0);

DropDITI(1,2,3,10,70,0);

GroupEnd();

Group("Initial Wash");

Wash(1,1,0,1,2,"10",500,"4.0",500,10,70,30,1,0,1000,0);

Wash(1,1,0,1,2,"5.0",500,"4.0",500,10,70,30,0,0,1000,0);

GroupEnd();

Group("Add UTP");

GetDITI2(1,"DiTi 50ul LiHa",0,0,10,70);

Mix(1,"CF wet contact","50",0,0,0,0,0,0,0,0,0,0,0,0,10,1,1,"06040080",10,0,0);

Aspirate(1,"CF wet contact","12",0,0,0,0,0,0,0,0,0,0,0,0,10,1,1,"06040080",0,0);

Dispense(1,"CF wet contact","12",0,0,0,0,0,0,0,0,0,0,0,0,10,2,1,"06041000",1,"3 rounds",0,1,0);

DropDITI(1,2,3,10,70,0);

GroupEnd();

Group("Initial Wash");

Wash(1,1,0,1,2,"10",500,"4.0",500,10,70,30,1,0,1000,0);

Wash(1,1,0,1,2,"5.0",500,"4.0",500,10,70,30,0,0,1000,0);
```

```
GroupEnd();

Group("Add IPTG");

GetDITI2(1,"DiTi 50ul LiHa",0,0,10,70);

Mix(1,"CF wet contact","50",0,0,0,0,0,0,0,0,0,0,0,0,10,1,1,"06040001",10,0,0);

Aspirate(1,"CF wet contact","15",0,0,0,0,0,0,0,0,0,0,0,0,10,1,1,"06040001",0,0);

Dispense(1,"CF wet contact","15",0,0,0,0,0,0,0,0,0,0,0,0,10,2,1,"06041000",1,"3 rounds",0,1,0);

DropDITI(1,2,3,10,70,0);

GroupEnd();

Group("Initial Wash");

Wash(1,1,0,1,2,"10",500,"4.0",500,10,70,30,1,0,1000,0);

Wash(1,1,0,1,2,"5.0",500,"4.0",500,10,70,30,0,0,1000,0);

GroupEnd();

Group("Add Sucrose");

GetDITI2(1,"DiTi 200ul LiHa",0,0,10,70);

Mix(1,">> SUCROSE 2M Wet contact DiTi <<        897","30",0,0,0,0,0,0,0,0,0,0,0,0,10,1,1,"06048000",5,0,0);

Aspirate(1,"SUCROSE 2M Wet contact DiTi","150",0,0,0,0,0,0,0,0,0,0,0,0,10,1,1,"06048000",0,0);

Dispense(1,"SUCROSE 2M Wet contact DiTi","150",0,0,0,0,0,0,0,0,0,0,0,0,10,2,1,"06041000",1,"3 rounds",0,1,0);

DropDITI(1,2,3,10,70,0);

GroupEnd();

Group("Initial Wash");

Wash(1,1,0,1,2,"10",500,"4.0",500,10,70,30,1,0,1000,0);

Wash(1,1,0,1,2,"5.0",500,"4.0",500,10,70,30,0,0,1000,0);
```

```
GroupEnd();

Group("mixing and we done");

GetDITI2(1,"DiTi 50ul LiHa",0,0,10,70);

Mix(1,">> PBS Dispense <<     713","50",0,0,0,0,0,0,0,0,0,0,0,10,2,1,"06041000",10,1,"3 rounds",0,1,0);

Mix(1,">> PBS Dispense <<     714","50",0,0,0,0,0,0,0,0,0,0,0,10,2,1,"06041000",10,1,"3 rounds",0,1,0);

Mix(1,">> PBS Dispense <<     715","50",0,0,0,0,0,0,0,0,0,0,0,10,2,1,"06041000",10,1,"3 rounds",0,1,0);

DropDITI(1,2,3,10,70,0);

GroupEnd();

Group("Initial Wash");

Wash(1,1,0,1,2,"10",500,"4.0",500,10,70,30,1,0,1000,0);

Wash(1,1,0,1,2,"5.0",500,"4.0",500,10,70,30,0,0,1000,0);

GroupEnd();

EndLoop();

GroupEnd();
```

Script 2-Lipid mixture solution preparation script for a final volume of 100µl synthetic cell, according to the optimized protocol:

```
Group("Initialization");

Vector("Move Roma","17","2",0,1,2,2,0,0);

UserPrompt("check all the eppendorfs are open",0,-1);

UserPrompt("Wait for melting",0,300);

GroupEnd();

Group("Initial Wash");

Wash(1,1,0,1,2,"30.0",500,"4.0",500,10,70,30,1,0,1000,0);

Wash(1,1,0,1,2,"5.0",500,"4.0",500,10,70,30,0,0,1000,0);

GroupEnd();

Group("First lipid solution");

GetDITI2(1,"DiTi 200ul LiHa",0,0,10,70);

Mix(1,">> mineral oil mixing <<        928","50",0,0,0,0,0,0,0,0,0,0,0,10,1,1,"06041000",5,0,0);

Aspirate(1,"mineral oil 100 ","100",0,0,0,0,0,0,0,0,0,0,0,10,1,1,"06041000",0,0);

Dispense(1,">> mineral oil 100  <<      929","100",0,0,0,0,0,0,0,0,0,0,0,10,2,1,"06041000",0,0);

DropDITI(1,2,3,10,70,0);

GroupEnd();

Group("Wash");

Wash(1,1,0,1,2,"10",500,"4.0",500,10,70,30,1,0,1000,0);

Wash(1,1,0,1,2,"5.0",500,"4.0",500,10,70,30,0,0,1000,0);

GroupEnd();

Group("second lipid solution");
```

```
GetDITI2(1,"DiTi 200ul LiHa",0,0,10,70);

Mix(1,">> mineral oil mixing <<              930","50",0,0,0,0,0,0,0,0,0,0,0,10,1,1,"0604@000",5,0,0);

Aspirate(1,"mineral oil 100 ","100",0,0,0,0,0,0,0,0,0,0,0,10,1,1,"0604@000",0,0);

Dispense(1,">> mineral oil 100  <<         933","100",0,0,0,0,0,0,0,0,0,0,0,10,2,1,"06041000",0,0);

Mix(1,">> mineral oil mixing <<              934","100",0,0,0,0,0,0,0,0,0,0,0,10,2,1,"06041000",5,0,0);

DropDITI(1,2,3,10,70,0);

GroupEnd();

Group("Initial Wash");

DropDITI(1,2,3,10,70,0);

Wash(1,1,0,1,2,"30.0",500,"4.0",500,10,70,30,1,0,1000,0);

Wash(1,1,0,1,2,"5.0",500,"4.0",500,10,70,30,0,0,1000,0);

GroupEnd();
```

Script 3- Emulsification script for a final volume of 100µl synthetic cell, according to the optimized protocol:

```
Group("Initialization");

Vector("Move Roma","17","2",0,1,2,2,0,0);

UserPrompt("check all the eppendorfs are open",0,-1);

GroupEnd();

Group("Initial Wash");

Wash(1,1,0,1,2,"30.0",500,"4.0",500,10,70,30,1,0,1000,0);

Wash(1,1,0,1,2,"5.0",500,"4.0",500,10,70,30,0,0,1000,0);

GroupEnd();

Group("oil mixing");

GetDITI2(1,"DiTi 200ul LiHa",0,0,10,70);

Mix(1,"Mix Lipids - 400","200",0,0,0,0,0,0,0,0,0,0,0,10,1,1,"06041000",10,0,0);

DropDITI(1,2,3,10,70,0);

GroupEnd();

Group("Wash");

Wash(1,1,0,1,2,"10",500,"4.0",500,10,70,30,1,0,1000,0);

Wash(1,1,0,1,2,"5.0",500,"4.0",500,10,70,30,0,0,1000,0);

GroupEnd();

Group("CF mixing and transfering");

GetDITI2(1,"DiTi 200ul LiHa",0,0,10,70);

Mix(1,">> PEG dispense <<     768","50",0,0,0,0,0,0,0,0,0,0,0,10,1,1,"0604@000",15,0,0);

Aspirate(1,"PEG dispense","100",0,0,0,0,0,0,0,0,0,0,0,10,1,1,"0604@000",0,0);
```

```
Dispense(1,">> PEG dispense <<            826","100",0,0,0,0,0,0,0,0,0,0,0,10,1,1,"06041000",0,0);
GroupEnd();
Group("Pipitaion");
BeginLoop("4","pipitation");
Aspirate(1,">> Lipid transfer 120 <<       820","150",0,0,0,0,0,0,0,0,0,0,0,10,1,1,"06041000",0,0);
Dispense(1,">> Lipid transfer 120 <<      816","150",0,0,0,0,0,0,0,0,0,0,0,10,1,1,"06041000",0,0);
Mix(1,">> Mix Lipids - 400 << 848","50",0,0,0,0,0,0,0,0,0,0,0,10,1,1,"06041000",30,0,0);
Aspirate(1,">> Lipid transfer 120 <<       821","150",0,0,0,0,0,0,0,0,0,0,0,10,1,1,"06041000",0,0);
Dispense(1,">> Lipid transfer 120 <<      798","150",0,0,0,0,0,0,0,0,0,0,0,10,1,1,"06041000",0,0);
Mix(1,">> Mix Lipids - 400 << 849","50",0,0,0,0,0,0,0,0,0,0,0,10,1,1,"06041000",30,0,0);
Aspirate(1,">> Lipid transfer 120 <<       825","150",0,0,0,0,0,0,0,0,0,0,0,10,1,1,"06041000",0,0);
Dispense(1,">> Lipid transfer 120 <<      801","150",0,0,0,0,0,0,0,0,0,0,0,10,1,1,"06041000",0,0);
Mix(1,">> Mix Lipids - 400 << 850","50",0,0,0,0,0,0,0,0,0,0,0,10,1,1,"06041000",30,0,0);
Aspirate(1,">> Lipid transfer 120 <<       823","150",0,0,0,0,0,0,0,0,0,0,0,10,1,1,"06041000",0,0);
Dispense(1,">> Lipid transfer 120 <<      804","150",0,0,0,0,0,0,0,0,0,0,0,10,1,1,"06041000",0,0);
Mix(1,">> Mix Lipids - 400 << 851","50",0,0,0,0,0,0,0,0,0,0,0,10,1,1,"06041000",30,0,0);
Aspirate(1,">> Lipid transfer 120 <<       824","150",0,0,0,0,0,0,0,0,0,0,0,10,1,1,"06041000",0,0);
Dispense(1,">> Lipid transfer 120 <<      807","150",0,0,0,0,0,0,0,0,0,0,0,10,1,1,"06041000",0,0);
Mix(1,">> Mix Lipids - 400 << 852","50",0,0,0,0,0,0,0,0,0,0,0,10,1,1,"06041000",30,0,0);
EndLoop();
DropDITI(1,2,3,10,70,0);
GroupEnd();
```

```
Group("Wash");

Wash(1,1,0,1,2,"10",500,"4.0",500,10,70,30,1,0,1000,0);

Wash(1,1,0,1,2,"5.0",500,"4.0",500,10,70,30,0,0,1000,0);

GroupEnd();
```
