# Supplementary material for: Scaling Up Synthetic Cell Production Using Robotics and Machine Learning Toward Therapeutic Applications
Source: Adv Biol (Weinh). 2025 Mar 31;9(5):2400671. doi: 10.1002/adbi.202400671 (PMC12078883; doi:10.1002/adbi.202400671)
Supplement: Supplementary file 4 — Supporting Information [file ADBI-9-2400671-s003.pdf]

ADVANCED BIOLOGY

Supporting Information

for *Adv. Biology*, DOI 10.1002/adbi.202400671

Scaling Up Synthetic Cell Production Using Robotics and Machine Learning Toward Therapeutic Applications

Noga Sharf-Pauker, Ido Galil, Omer Kfir, Gal Chen, Rotem Menachem, Jeny Shklover, Avi Schroeder and Shanny Ackerman**

Supplementary 4:

Table S1: Successful and unsuccessful improvements in the SC production process

	**Successful improvements**	**Unsuccessful improvements**
**Preparation and storage of stock solutions**	- The CFPS pre-inner , with or without the DNA plasmid, and the feeding solutions do not need to be made fresh for every experiment, they can be stored at -20°C for a month (Figure 1C)).<br><br>- The *E. coli* S30 lysate leftover does not need to be discarded after thawing; it can be refrozen at -20°C up to three times without losing activity (Figure 1D).<br><br>- Polyethylene glycol 6000 (PEG6000) added to the CFPS inner solution can be incorporated at a final concentration of 1.5% w/v (instead of the previously used 3% w/v in earlier publications) to enhance CFPS activity without reducing the concentration of SCs (Supplementary 2 Figure S1).<br><br>- Liquid handler may be used to create the pre-inner and the feeding solutions, as well as the lipid mixture, in order to increase accuracy and reproducibility (Figure 2).<br><br>- Prepared SCs after protein expression can be stored at 4°C overnight (Supplementary 2 Figure S2).	- Complete CFPS solutions (containing *E.coli* S30 lysate) and prepared SCs cannot be stored prior to protein expression, as they lose activity after overnight storage at 4°C (Supplementary 2 Figure S2). Moreover, adding building blocks (e.g., nucleotides, amino acids, energy sources) to the SCs' feeding solution after overnight storage at 4°C does not enhance expression.
**Lipid amounts and composition**	- Reducing the lipid mixture ratio from 1:10 (CFPS solution to 10 mg/mL POPC and 10 mg/mL cholesterol in mineral oil) to 1:2 significantly decreased oil remnants without lowering the concentration of SCs (Figure 3A-C).	- A ratio of less than 1:2 between CFPS solution and lipid mixture solution resulted in a decrease in SCs concentration (Supplementary 2 Figure S3).
**Emulsification process and scale up**	- For CFPS solution up to 200μl, 30 seconds of pipetting and vortexing are sufficient. For CFPS solution in volumes between 200μl and 500μl, 1 minute of pipetting and vortexing is required (Figure 6). For CFPS solution in volumes between 500μl and 1000μl, 1.5 minute of pipetting and vortexing is required (Figure 6).<br><br>- A GentleMACS can be used to emulsify larger volumes of CFPS solution into the lipid mixture for volumes exceeding 500µL CFPS solution (Figure 6).	- Using only pipetting or only vortexing to manually create the emulsion is insufficient, leading to a reduction in SC concentration and an increase in SC aggregation (Supplementary 2 Figure S4).<br><br>- Emulsification by manual pipetting is not sufficient for higher volumes than 500μl and resulted in reduction in SCs concentration (Figure 6).

	- A liquid handler may be used for emulsification, but the process is slow and not efficient for volumes higher than 100μl (Figure 2).	- Manual emulsifier Multi-Gen 7XL is not suitable for SC emulsification (Supplementary 2 Figure S4)
**Particle size and aggregation reduction**	- Filters, such as cell strainers, with cut-off down to 10μm can be used to reduce the size and aggregation of SCs (Supplementary 2 Figure S5).	- Manual extruders, high pressure extruders and filters with cut-off lower than 10μm are unsuitable for reducing the size and aggregation of SCs without compromising their concentration or activity (Supplementary 2 Figure S5).
**Cost and material reduction**	- The reduction in lipid mixture volume mentioned above decreases the amounts of lipids and mineral oil required for SC production.<br><br>- 3-PGA can be omitted from the feeding solution without affecting the SCs activity	- The feeding solution cannot be replaced with a glucose solution without compromising SC activity (as shown in Supplementary 2 Figure S6)

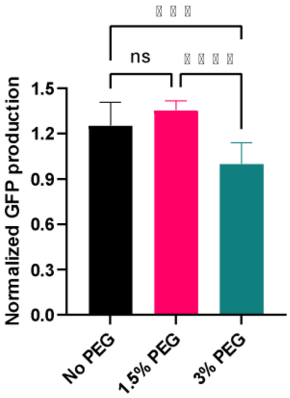

Figure S1: Activity of CFPS inner solutions with varying weight-to-volume percentages of PEG 6000. Data are expressed as mean ± s.e.m (n = 9 independent samples). All results were normalized to 3% PEG6000. Two-way ANOVA with adjusted P value in Tukey multiple comparisons tests, ***P=0.0002, ****P=<0.0001.

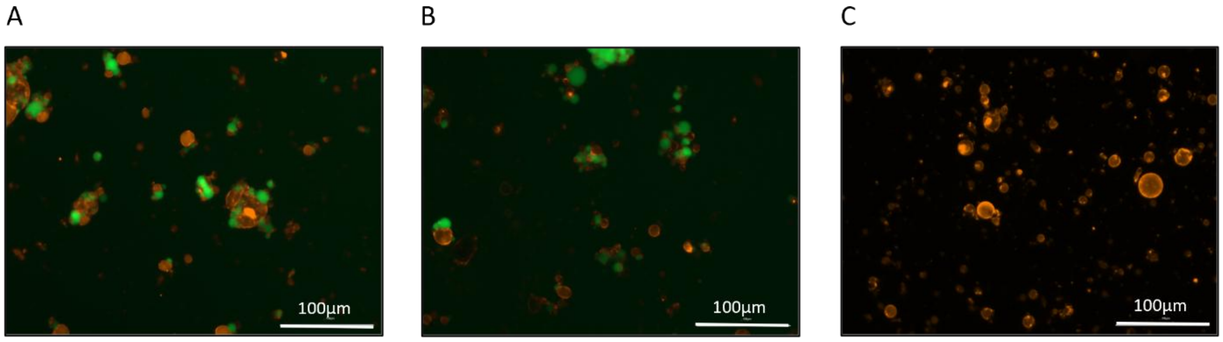

Figure S2: Representative images of sfGFP-producing SC stability overnight at 4◦C. (A) Fresh SCs after sfGFP expression for 2 hours at 37◦C. (B) SCs with sfGFP expression completed before being kept overnight at 4°C. (C) SCs with sfGFP expression completed after being kept overnight at 4°C. *Produced GFP (in green), Rhodamine –labeled membrane (in orange)*. The images were taken using a fluorescent microscope with RFP and GFP filters, merged.

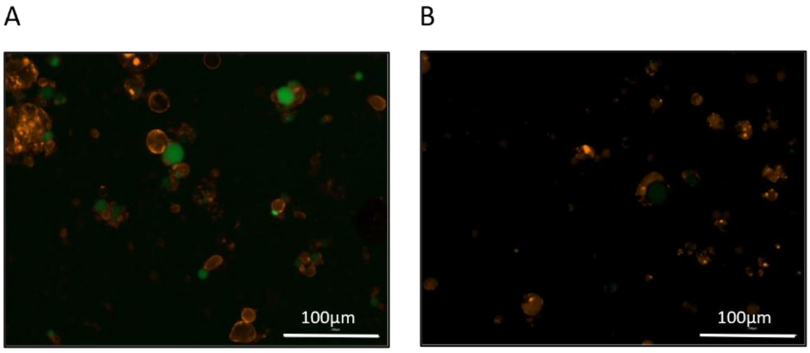

Figure S3: Representative images of sfGFP-producing SCs produced with different ratios of CFPS solution to lipid mix solution. (A) A ratio of 1:2 between CFPS solution and lipid mix solution. (B) A ratio of 1:1.5 between CFPS solution and lipid mix solution. *Produced GFP (in green), Rhodamine –labeled membrane (in orange).* The image was taken using a fluorescent microscope with RFP and GFP filters, merged.

A

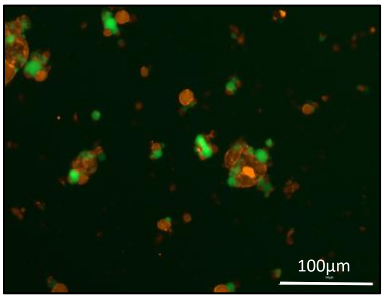

B

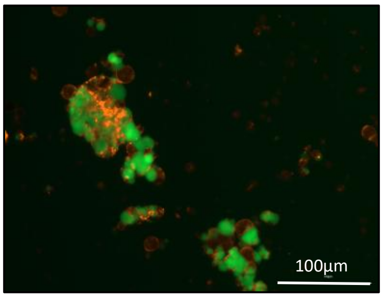

C

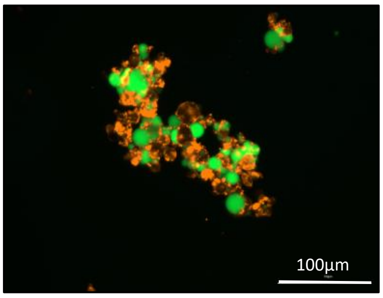

D

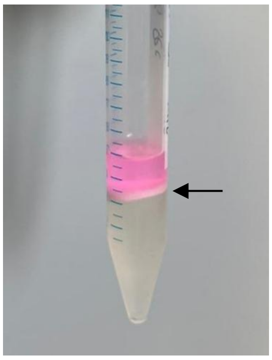

Figure S4: Representative images of sfGFP-producing SCs produced by emulsifying CFPS inner solution into the lipid mix solution using different emulsification methods. (A) Emulsification by both pipetting and vortexing. (B) Emulsification by pipetting (C) Emulsification by vortexing. (D) Emulsion created using a manual emulsifier (Multi-Gen 7XL Homogenizer) for 5 seconds at the lowest speed, followed by centrifugation at 100 rpm for 10 minutes and then 400 rpm for 10 minutes. For (A)-(C), *produced GFP (in green), Rhodamine –labeled membrane (in orange).* The images were taken using a fluorescent microscope with RFP and GFP filters, merged.

A

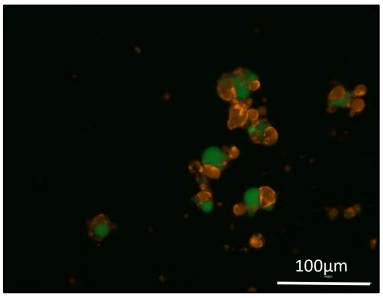

B

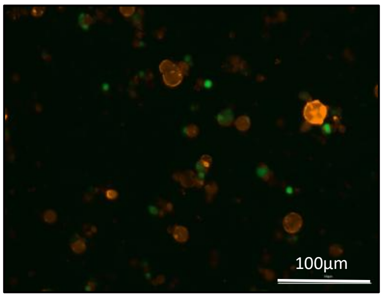

C

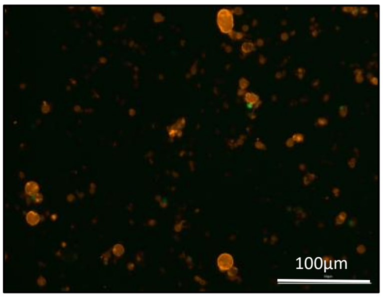

Figure S5: Representative images of sfGFP-producing SCs before and after passing through filters. (A) Original size and aggregation of SCs, without passing through a filter. (B) SCs after passing through a cell strainer with a 10µm cut-off (C) SCs after passing through a cell strainer with a 5µm cut-off. *Produced GFP (in green), Rhodamine –labeled membrane (in orange).* The images were taken using a fluorescent microscope with RFP and GFP filters, merged.

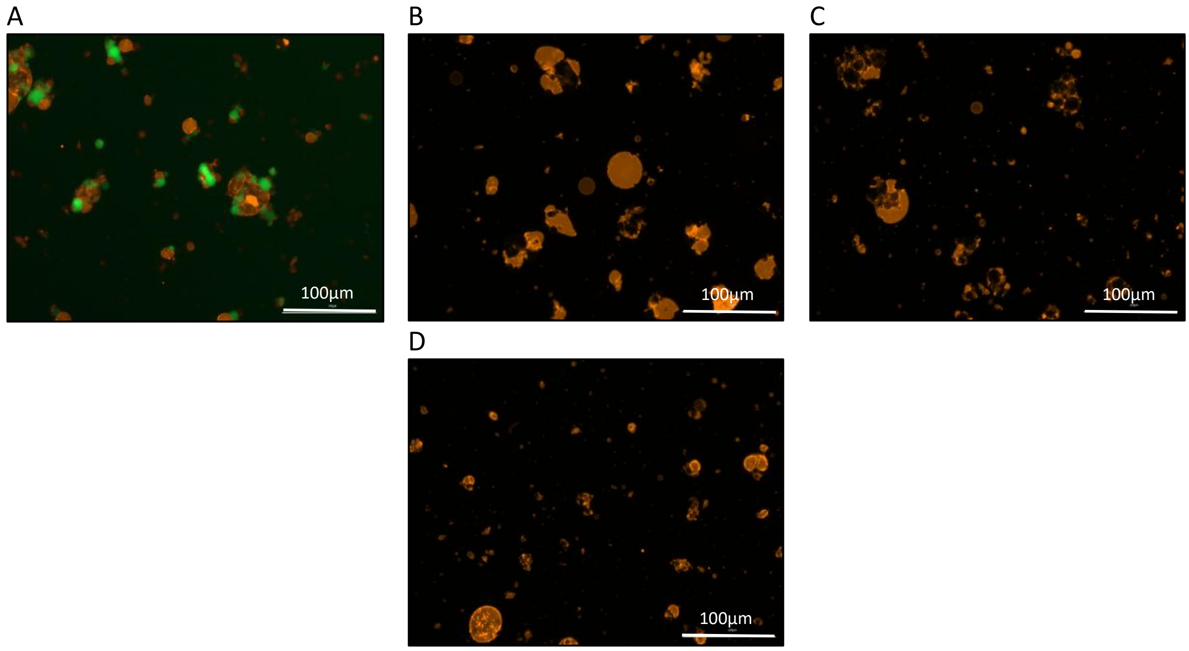

Figure S6: Representative images of sfGFP-producing SCs resuspended with different solutions. (A) SCs resuspended using feeding solution. (B)-(D) SCs resuspended using glucose solution in concentrations of 100,500,1200mM respectively. *Produced GFP (in green), Rhodamine –labeled membrane (in orange).* The images were taken using a fluorescent microscope with RFP and GFP filters, merged.
